# Sarcopenia and cardiovascular diseases: A systematic review and meta‐analysis

**DOI:** 10.1002/jcsm.13221

**Published:** 2023-04-01

**Authors:** Xinrong Zuo, Xuehong Li, Kuo Tang, Rui Zhao, Minming Wu, Yang Wang, Tao Li

**Affiliations:** ^1^ Department of Anesthesiology The Affiliated Hospital of Southwest Medical University Luzhou Sichuan China; ^2^ Department of Anesthesiology, Laboratory of Mitochondria and Metabolism, National Clinical Research Center for Geriatrics West China Hospital of Sichuan University Chengdu Sichuan China

**Keywords:** cardiac arrhythmia, cardiovascular diseases, coronary artery disease, heart failure, prevalence, sarcopenia

## Abstract

Sarcopenia is an age‐related disease and is often accompanied by other diseases. Now, many studies have shown that cardiovascular diseases (CVDs) may raise the incidence rate of sarcopenia. Therefore, the purpose of this study was to conduct a systematic review and meta‐analysis to investigate the prevalence of sarcopenia in patients with CVDs compared with the general population, defined as relatively healthy non‐hospitalized subjects. The databases of PubMed, Embase, Medline and Web of Science were searched for eligible studies published up to 12 November 2022. Two assessment tools were used to evaluate study quality and the risk of bias. Statistical analysis was conducted using STATA 14.0 and R Version 4.1.2. Thirty‐eight out of the 89 629 articles retrieved were included in our review. The prevalence of sarcopenia ranged from 10.1% to 68.9% in patients with CVDs, and the pooled prevalence was 35% (95% confidence interval [95% CI]: 28–42%). The pooled prevalence of sarcopenia was 32% (95% CI: 23–41%) in patients with chronic heart failure (CHF), 61% (95% CI: 49–72%) in patients with acute decompensated heart failure (ADHF), 43% (95% CI: 2–85%) in patients with coronary artery disease, 30% (95% CI: 25–35%) in patients with cardiac arrhythmia (CA), 35% (95% CI: 10–59%) in patients with congenital heart disease and 12% (95% CI: 7–17%) in patients with unclassed CVDs. However, in the general population, the prevalence of sarcopenia varied from 2.9% to 28.6% and the pooled prevalence was 13% (95% CI: 9–17%), suggesting that the prevalence of sarcopenia in patients with CVDs was about twice compared with the general population. The prevalence of sarcopenia was significantly higher only in patients with ADHF, CHF and CA compared with the general population. There is a positive correlation between CVDs and sarcopenia. The prevalence of sarcopenia is higher in patients with CVDs than that in the general population. With global aging, sarcopenia has brought a heavy burden to individuals and society. Therefore, it is important to identify the populations with high‐risk or probable sarcopenia in order to do an early intervention, such as exercise, to counteract or slow down the progress of sarcopenia.

## Introduction

Sarcopenia is a geriatric syndrome characterized by progressive muscle wasting and dysfunction, which leads to shorter life expectancy and lower life quality. In 1989, Rosenberg first described this syndrome as muscle loss distinct from cachexia, simple starvation or end‐stage disease complications.^1^ There are many risk factors for sarcopenia, such as malnutrition, sedentary lifestyle, chronic diseases and iatrogenic factors. Sarcopenia involves many complex and interactive pathophysiological mechanisms, including inflammation, mitochondrial dysfunction, oxidative stress, hormonal changes, synthetic metabolic resistance, lipotoxicity and microvascular changes.^2^ In 2016, sarcopenia was officially identified as a muscle disease and its International Classification of Diseases, Tenth Revision, Clinical Modification (ICD‐10‐CM) code is M62.84.^3^ Mounting evidence shows that sarcopenia is closely related to multiple adverse outcomes, such as frailty, fall or even fracture, physical functional decline, recognition functional deterioration, the significantly increased incidence rate of morbidity and mortality, and substantial healthcare costs incurred by the increasing life expectancy and increasing aging population worldwide, all of which seriously affect the life quality of the elderly.

Cardiovascular diseases (CVDs) place a heavy burden on patients and their families as well as healthcare systems and remain a major cause of mortality and disability worldwide, accounting for one third of global deaths. In 2019, 6.2 million people aged 30–70 died from CVDs, whereas the total number of deaths caused by CVDs increased by 34.9% from 1990 to 2019.^4^ Cardiac metabolism, behaviour, environment, genetic factors and socio‐economic and psychosocial risk factors are major causes of CVDs.^4^, ^5^

As common age‐related diseases, sarcopenia and CVDs have become the major concern of public health. With the gradual development and renewal of the consensus on sarcopenia, more and more studies have discovered the relationship between sarcopenia and CVDs, such as heart failure (HF), ischaemic heart disease (IHD), coronary artery disease (CAD), congenital heart disease (CHD) and cardiac arrhythmia (CA).^6^, ^7^, ^8^, ^9^, ^10^, ^11^, ^12^, ^13^, ^14^, ^15^ Sarcopenia and CVDs share several common risk factors, such as lack of physical activity, up‐regulation of inflammatory factors, mitochondrial dysfunction, oxidative stress, hormone changes and low muscle blood.^16^, ^17^ The deficiency and deterioration of cardiac function affect the blood flow and metabolism of the myocardium and skeletal muscle. Skeletal muscle is one of the important metabolic and endocrine organs, and thus, skeletal muscle atrophy caused by sarcopenia will aggravate CVDs. It is a reasonable inference that certain relationships exist between sarcopenia and CVDs.

At present, some studies have focused on the pathological relationship between sarcopenia and CVDs. However, due to the disparities in diagnostic criteria for sarcopenia, patient characteristics and the types of CVDs, the prevalence of sarcopenia in patients with CVDs varies (10.1–68.9%). Therefore, we conducted this review and meta‐analysis to evaluate and compare the convincing evidence on the prevalence of sarcopenia in patients with CVDs and whether CVDs would increase the incidence of sarcopenia.

## Methods

### Databases and search strategy

The Preferred Reporting Items for Systematic Reviews and Meta‐Analyses 2020 statement (PRISMA 2020)^18^ principles were followed in this systematic review. Two reviewers systematically searched all relevant articles published up to 12 November 2022 on the databases of PubMed, Embase, Medline and Web of Science and screened the citations included in such articles. CVDs were searched, such as HF, CAD, IHD, CHD, cardiomyopathy, valvular heart disease, rheumatic heart disease, pulmonary heart disease, aortic aneurysm and CA. The subtypes of CVDs associated with sarcopenia were finally selected, including HF, CAD, CHD, CA and unclassed CVDs. Sarcopenia and its prevalence were also searched. The citations included in the articles found in the aforementioned database search for additional related studies were also screened. The detailed search strategies can be found in *Table* *S1*.

### Protocol and registration

The review protocol is registered in PROSPERO (CRD42022326401).

### Inclusion and exclusion criteria

All included studies related to the prevalence of sarcopenia in patients with CVDs met the following criteria: (1) cross‐sectional or prospective cohort studies; (2) population: ≥18 years old, patients with HF, CAD, CHD, CA or unclassed CVDs hospitalized or diagnosed according to the clinical gold standard; (3) sarcopenia assessment must be based on the consensus criteria of sarcopenia (comprehensive: low muscle mass [LMM] and low muscle strength [LMS] and/or low physical performance [LPP] were combined; non‐comprehensive: one of the three aforementioned aspects was applicable respectively); (4) lean skeletal muscle mass was detected with instruments commonly used previously to the study, such as dual‐energy X‐ray absorptiometry (DXA), bioelectrical impedance analysis (BIA), magnetic resonance imaging and computed tomography; and (5) provision of prevalence data for sarcopenia (primary outcome) in patients with CVDs subtypes. The inclusion criteria of the sarcopenia prevalence in the general population, defined as relatively healthy non‐hospitalized subjects, were as follows: (1) cross‐sectional or prospective cohort studies; (2) population: relatively healthy non‐hospitalized population, excluding people with acute injury such as joint trauma or surgery, people who take supplements capable of modifying body composition (e.g., corticosteroids and anabolic hormones) and people with chronic diseases, such as CVDs, chronic liver/lung/kidney disease, type I or II diabetes, hypertension, metabolic syndrome, neurological or musculoskeletal diseases, mental diseases (e.g., dementia, anxiety and depression), cancer and other metabolic or inflammatory diseases; (3) assessment method of sarcopenia in the general population is similar to that of patients with CVDs; and (4) provision of prevalence data for sarcopenia (primary outcome) in the general population.

Studies were excluded according to the following criteria: (1) sarcopenia diagnostic criteria or prevalence were not reported; (2) study subjects were not CVDs; and (3) non‐original research, such as reviews, case reports, editorials, letters to editors, comments, conference abstracts and animal studies. Similarly, the exclusion criteria for studies on the general population were as follows: (1) sarcopenia diagnostic criteria or prevalence were not reported; (2) study population with acute/chronic diseases or taking drugs/supplements that may or may not interfere with skeletal muscle quality and function; and (3) non‐original research, such as reviews, case reports, editorials, letters to editors, comments, conference abstracts and animal studies.

### Study selection

All searched articles were imported into EndNote for repeated inspection. After deleting duplicate studies, two independent reviewers screened the titles and abstracts of all the articles and selected potential relevant studies. Then two co‐authors separately reviewed the full texts of these studies and, according to the inclusion/exclusion criteria, decided on the final studies and relevant references to be included in the review. The disagreement between the two co‐authors was resolved by discussion with the third author.

### Data extraction

We used a predefined data collection form (*Table* *1*) to extract information, including first author, publication year, country, region, population setting, sample size, sex ratio, age, subtypes of CVDs, the prevalence of sarcopenia, diagnostic methods of sarcopenia, assessment of handgrip strength and diagnostic criteria of sarcopenia. The main outcome of interest was the prevalence of sarcopenia in patients with CVDs. We extracted similar information from studies on the general population, including first author, publication year, country, region, population setting, sample size, sex, age, the prevalence and measurement methods of sarcopenia, cut‐off values of LMS and diagnostic criteria of sarcopenia (*Table* *S2*).

**TABLE 1:** Characteristics of the included studies and main outcomes of sarcopenia and CVDs

First author and year	Country	Study region	Population setting	Sample size	Male, *n* (%)	Age^a^ (years)	CVDs	Prevalence of sarcopenia			Assessment method	HGS measure	Sarcopenia diagnostic criteria
								Total *n* (%)	Male *n* (%)	Female *n* (%)			
Zhao (2020)	China	Asia	Hospitalized	355	207 (58.3)	71	CHF	198 (55.8)	105 (50.7)	93 (62.8)	LMM (BIA) + LMS (HGS) + LPP (4mGS)	Dominant hand	AWGS (2014)
Fülster (2012)	Germany	Europe	Ambulatory	200	159 (79.5)	66.9	CHF	39 (19.5)	37 (23.3)	2 (4.9)	LMM (DXA) and LPP (4mGS)	Hands	ICS (2011)
Bekfani (2016)	Germany England Slovenia	Europe	Ambulatory	117	79 (67.5)	69.8	HFpEF	23 (19.7)	20 (25.3)	3 (7.9)	LMM (DXA)	Hands	ICS (2011)
Hu (2018)	China	Asia	Hospitalized	182	108 (59.3)	77.5	CHF	75 (41.2)	48 (44.4)	27 (36.5)	LMM (DXA) + LMS (HGS) + LPP (6mGS)	Dominant hand	AWGS (2014)
Konishi (2020)	Japan	Asia	Hospitalized	942	550 (58.4)	79	HF	187 (19.8)	132 (24.0)	55 (14.0)	LMM (BIA) + LMS (HGS) + LPP (4mGS)	Both hands	AWGS (2014)
Tsuchida (2018)	Japan	Asia	Hospitalized	38	25 (65.8)	75	ADHF	20 (52.6)	16 (64.0)	4 (30.8)	LMM (DXA)	NA	Other^b^
Eschalier (2020)	France	Europe	Hospitalized	140	82 (58.6)	75.8	ADHF	91 (65.0)	54 (65.9)	37 (63.8)	LMM (BIA) + LMS (HGS) + LPP (4mGS)	Dominant hand	EWGSOP (2010)
Dos Santos (2016)	Germany	Europe	Ambulatory	228	181 (79.4)	68.8	CHF	37 (16.2)	36 (19.9)	1 (2.1)	LMM (DXA)	NA	Other^b^
Fonseca (2020)	Brazil	South America	Ambulatory	168	168 (100)	58	HFrEF	66 (39.3)	66 (39.3)	0	LMM (DXA)	Dominant hand	EWGSOP (2010)
Canteri (2019)	Brazil	South America	Ambulatory	79	46 (58.2)	65.6	HFrEF	8 (10.1)	7 (15.2)	1 (3.0)	LMM (DXA) + LPP (4mGS)	Both hands	EWGSOP2 (2018)
Fonseca (2019)	Brazil	South America	Ambulatory	116	116 (100)	55	CHF	33 (28.0)	33 (28.0)	0	LMM (DXA) + LMS (HGS)	Dominant hand	EWGSOP (2010)
Kono (2020)	Japan	Asia	Hospitalized	186	81 (43.5)	79.8	CHF	77 (41.3)	15 (18.5)	62 (59.0)	LMS, LPP, BMI < 18.5 (any two)	Hands	Other^b^
Hajahmadi (2017)	Iran	Asia	Ambulatory	55	32 (58.2)	37.3	CHF	26 (47.3)	18 (56.3)	8 (34.9)	LMM (DXA)	NA	Other^b^
Onoue (2016)	Japan	Asia	Hospitalized	119	73 (61.0)	76.1	CHF	82 (68.9)	53 (72.6)	29 (63.0)	Sarcopenia score	Dominant hand	Other^b^
Emami (2018)	Germany	Europe	Ambulatory	207	207 (100)	67.3	CHF	30 (14.5)	30 (14.5)	0	LMM (DXA)	Hands	ICS (2011)
Zhang (2019)	China	Asia	Hospitalized	364	218 (59.9)	74.6	CAD	81 (23.3)	35 (43.2)	46 (28.0)	LMM (BIA) + LMS (HGS) + LPP (6mGS)	Both hands	AWGS (2014)
Santana (2019)	Brazil	South America	Hospitalized	99	49 (49.5)	71.6	CAD	64 (64.6)	38 (76.0)	26 (53.1)	LMM (BIA) + LMS (HGS) + LPP (4mGS)	Hands	Other^b^
Heshmat (2021)	Iran	Asia	Urban population	354	177 (50.0)	69.3	CA	106 (30.5)	NR	NR	LMM (DXA) + LMS (HGS)	Both hands	EWGSOP2 (2018)
Sandberg (2019)	Sweden	Europe	Hospitalized	73	51 (69.9)	35.8	CHD	37 (50.7)	24 (47.1)	13 (59.1)	LMM (DXA)	Dominant hand	EWGSOP (2010)
Tran (2020)	Australia	Oceania	Hospitalized	28	13 (46.4)	26	CHD	11 (39.0)	NR	NR	LMM (DXA)	Both hands	Other^b^
Shiina (2019)	Japan	Asia	Ambulatory	117	50 (42.7)	36.9	CHD	19 (16.2)	7 (14.0)	12 (18.0)	LMM (BIA)	NA	Other^b^
Sasaki (2020)	Japan	Asia	Hospitalized	160	100 (62.5)	67	CVDs	19 (11.9)	8 (8.0)	11 (16.4)	LMM (BIA) + LMS (HGS) + LPP (4mGS)	Both hands	AWGS (2014)

Abbreviations: ADHF, acute decompensated heart failure; AWGS, Asian Working Group for Sarcopenia; BIA, bioelectrical impedance analysis; BMI, body mass index; CA, cardiac arrhythmia; CAD, coronary artery disease; CHD, congenital heart disease; CHF, chronic heart failure; CVDs, cardiovascular diseases; DXA, dual‐energy X‐ray absorptiometry; EWGSOP, European Working Group on Sarcopenia in Older People; GS, gait speed; HFpEF, heart failure with preserved ejection fraction; HFrEF, heart failure with reduced ejection fraction; HGS, handgrip strength; ICS, International Consensus on Sarcopenia; LMM, lower muscle mass; LMS, lower muscle strength; LPP, lower physical performance; NA, not applicable; NR, not reported.

^a^ Mean as reported.

^b^ Sarcopenia diagnostic criteria other than the EWGSOP (2010), EWGSOP2 (2018), AWGS (2014), AWGS (2019), Foundation for the National Institutes of Health (FNIH) and International Working Group on Sarcopenia (IWGS, 2011).

### Assessment of methodological quality

The methodological quality of each study was independently assessed by two researchers using the National Institutes of Health (NIH) Quality Assessment Tool for Observational Cohort and Cross‐Sectional Studies,^19^ which includes 14 items and assesses the quality of cohort and cross‐sectional studies. In addition, we adopted a specific tool consisting of 10 items^20^ related to measurement, selection and analysis, to assess the risk of bias in prevalence studies with high reliability and consistency. The bias risk score was defined as follows: low risk ≥ 8; moderate risk = 6–7; and high risk ≤ 5. Similarly, disagreements were resolved by discussing with the third author.

### Statistical analyses

A random‐effects model was used to evaluate the pooled prevalence of sarcopenia in patients with different types of CVDs in this meta‐analysis to produce a lower type I error rate. A forest plot was generated using STATA 14 (Version 14.0, StataCorp, College Station, TX, USA) and R Version 4.1.2, describing the pooled incidence of sarcopenia and the corresponding 95% confidence interval (95% CI) for each study and overall estimate. Based on the statistical test of *I* ^2^, the *Q*‐test was used to estimate the heterogeneity between studies. A *P* value < 0.10 or an *I* ^2^ value > 50% indicated statistical heterogeneity. To investigate potential causes of heterogeneity, we conducted subgroup analyses by subtype of CVDs, study region, population background, gender and evaluation method of sarcopenia. Sensitivity analyses were performed by excluding each study to assess the stability of the study. Egger's test and Begg's test were designed to assess publication bias.

## Results

### Search process

**FIGURE 1:**
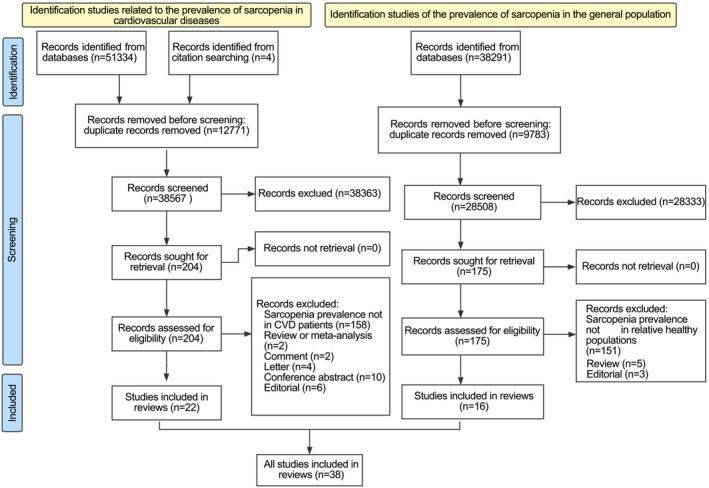
The flow chart of the literature selection in patients with cardiovascular diseases (CVDs) and the general population.

A total of 51 338 articles related to sarcopenia and CVDs were found in the databases after the systematic search. After duplicates were deleted, the titles and abstracts of 38 567 articles were screened and read, and 38 363 irrelevant studies were removed. A total of 204 relevant studies were submitted for full‐text screening, of which 182 were excluded. The reasons for excluding certain articles can be found in the flow chart. Finally, 22 articles^6^, ^7^, ^8^, ^9^, ^10^, ^11^, ^12^, ^21^, ^22^, ^23^, ^24^, ^25^, ^26^, ^27^, ^28^, ^29^, ^30^, ^31^, ^32^, ^33^, ^34^, ^35^ involving 4327 patients were included in our review. In addition, we searched 38 291 articles about the prevalence of sarcopenia in the general population. A total of 175 relevant studies were submitted for full‐text screening and included 16 articles in our review (*Figure* *1*).

### Study characteristics

The main characteristics of the included CVDs studies investigating prevalence are summarized in *Table* *1*. Among the 22 eligible studies published between 2012 and 2021, there were 15 studies involving HF, 2 involving CAD, 3 involving CHD, 1 involving CA and 1 involving unclassed CVDs. A total of 4327 individuals were enrolled, and over half of them were male (*n* = 2772). The mean age of study participants ranged from 26 to 79.8 years. Twelve studies were involved with inpatients, nine with outpatients and one with the urban population. Eleven of the selected studies used non‐comprehensive and 11 studies used comprehensive definitions of sarcopenia. The selected studies were from different regions, including 11 studies in Asia, 4 in South America, 6 in Europe and 1 in Oceania. The characteristics of the studies investigating sarcopenia prevalence in the general population can be found in *Table* *S2*, demonstrating that a total of 9541 individuals were enrolled among the 16 studies. The prevalence of sarcopenia varied from 2.9% to 28.6%. The report showed that nine studies were conducted in Asia, five in Europe, one in South America and one in North America. These articles had slightly different definitions of sarcopenia. Among them, 13 studies used comprehensive diagnostic methods, 2 studies used LMM and only 1 assessed sarcopenia using LMS.

### Risk of bias in the included studies

The overall quality of included studies was moderate when assessed by the NIH Quality Assessment Tool for Observational Cohort and Cross‐Sectional Studies (details in *Table* *S3*). In addition, when evaluated by prevalence study assessment, 12 studies were classed as moderate risk of bias and 22 as low risk of bias (*Table* *S4*).

### Diagnostic methods for sarcopenia

*Table* *2* summarizes each study's detailed diagnostic criteria and cut‐off values in sarcopenia and CVDs. Of the 22 studies, 7 studies used comprehensive criteria by combining LMM, LMS and LPP to diagnose sarcopenia, whereas 2 studies used LMM + LMS and 2 studies used LMM + LPP to detect sarcopenia. Other studies utilized non‐comprehensive criteria. Skeletal muscle mass was measured using BIA (7 studies), DXA (13 studies), calf circumference (1 study) and body mass index (BMI) (1 study). Muscle strength was measured with a handgrip dynamometer, and physical performance was measured with gait speed tests (4, 4.57, 6 and 10 m) and a 6‐min walk test. One study defined sarcopenia through sarcopenia scores and formulas involving age, calf circumference and grip strength. The detailed diagnostic criteria and cut‐off values of each study were summarized in *Table* *S5*, involving the prevalence of sarcopenia in the general population. Of the 16 studies, 13 used comprehensive criteria by combining LMM, LMS and LPP to diagnose sarcopenia, whereas 2 studies used LMM and 1 study used LMS to diagnose sarcopenia. Skeletal muscle mass was measured using BIA (six studies), DXA (eight studies) and equation (one study). Muscle strength was measured with a handgrip dynamometer, and physical performance was measured with gait speed tests (4, 5, 6 and 10 m).

**TABLE 2:** The details of diagnostic criteria and cut‐off points of each study

	Diagnosis criteria	References
**Low muscle mass**		
BIA	AWGS (2014)^36^ or AWGS (2019)^37^: SMI < 7.0 kg/m^2^ for men and SMI < 5.7 kg/m^2^ for women	Zhao (2020), Zhang (2019), Sasaki (2020) and Konishi (2020)
	Kuczmarski et al. (2000)^38^ and Janssen et al. (2004)^39^: SMI < 10.76 kg/m^2^ for men and SMI < 6.76 kg/m^2^ for women	Santana (2019)
	Morley et al. (2001)^40^: SMI < 7.0 kg/m^2^ in men and SMI < 5.7 kg/m^2^ in women	Shiina (2019)
	EWGSOP (2010)^41^: SMI < 10.75 kg/m^2^ for men and SMI = 6.75 kg/m^2^ for women	Eschalier (2020)
DXA	EWGSOP2 (2018)^42^: SMI < 7.0 kg/m^2^ for men and SMI < 5.4 kg/m^2^ for women	Heshmat (2021)
	AWGS (2014)^36^: SMI < 7.0 kg/m^2^ for men and SMI < 5.4 kg/m^2^ for women	Hu (2018)
	EWGSOP (2010)^41^: The 10th percentile of the residuals of the regression	Sandberg (2019)
	Morley et al. (2001)^40^: Appendicular skeletal muscle mass (ASMM) 2 standard deviations (SDs) below the mean of a young healthy reference group aged 18–40 years	Dos Santos (2016)
	EWGSOP (2010) and Baumgartner et al. (1998)^43^: SMI 2 or more SDs below the mean for healthy, young Japanese subjects (<6.87 kg/m^2^ in males and <5.46 kg/m^2^ in females)	Tsuchida (2018)
	ICS (2011)^44^: Muscle mass 2 SDs below the mean of a healthy young reference group aged 18–40 years	Emami (2018), Fülster (2012) and Bekfani (2016)
	Buckinx et al. (2018)^45^: ALMI *Z* score: −2 or lower	Tran (2020)
	EWGSOP (2010) and Baumgartner et al. (1998)^43^: SMI < the 20th percentile (7.03 kg/m^2^) Newman et al. (2003)^46^: The 20th percentile of the residuals of the regression	Fonseca (2020)
	FNIH (2014) and Studenski et al. (2014)^47^: ALM/BMI < 0.789 in men and ALM/BMI < 0.512 in women	Canteri (2019)
	EWGSOP (2010) and Baumgartner et al. (1998)^43^: SMI < 7.26 kg/m^2^	Fonseca (2019)
	EWGSOP (2010)^41^: SMI < 7.26 kg/m^2^ in men and SMI = 5.45 kg/m^2^ in women	Hajahmadi (2017)
BMI	The Japanese Geriatric Society^24^: BMI < 18.5 kg/m^2^	Kono (2020)
**Low muscle strength**		
HGS	AWGS (2014)^36^: <26 kg for men and <18 kg for women	Zhao (2020), Hu (2018), Zhang (2019), Sasaki (2020) and Konishi (2020)
	EWGSOP (2010)^41^: <30 kg for men and 20 kg for women	Eschalier (2020) and Fonseca (2019)
	EWGSOP (2018)^42^: <26 kg for men and <18 kg for women	Heshmat (2021)
	Lauretani et al. (2003)^48^: <20 kg for men and <30 kg for women	Santana (2019)
	Wong (2016)^49^: <86 ± 20% of reference values	Tran (2020)
	EWGSOP (2010)^41^: <30 kg for men and <20 kg for women, lower the 20th percentile	Fonseca (2020)
	Dam et al. (2014)^50^: <26 kg in men and <16 kg in women	Canteri (2019)
	The Japanese Geriatric Society^24^: <26 kg in males or <18 kg in females	Kono (2020)
**Low physical performance**		
4mGS	AWGS (2014)^36^: GS < 0.8 m/s	Zhao (2020) and Konishi (2020)
	Abellan van Kan et al. (2011)^51^: GS < 0.8 m/s	Santana (2019)
	EWGSOP (2010)^41^: GS < 0.8 m/s	Eschalier (2020) and Canteri (2019)
	ICS (2011)^44^: GS < 1.0 m/s	Fülster (2012)
4.57mGS	EWGSOP2 (2018)^42^: GS < 0.8 m/s	Heshmat (2021)
6mGS	AWGS (2014)^36^: GS < 0.8 m/s	Hu (2018) and Zhang (2019)
10mGS	AWGS (2014)^36^: GS < 0.8 m/s	Sasaki (2020)
	The Japanese Geriatric Society^24^: GS < 0.8 m/s	Kono (2020)
6‐min WT	ICS (2011)^44^: <400 m	Dos Santos (2016) and Bekfani (2016)
**Others**		
Sarcopenia score	Ishii et al. (2014)^52^: ≥105 in men and ≥120 in women	Onoue (2016)

Abbreviations: 6‐min WT, 6‐min walk test; ALM, appendicular lean mass; ALMI, appendicular lean mass index; AWGS, Asian Working Group for Sarcopenia; BIA, bioelectrical impedance analysis; BMI, body mass index; DXA, dual‐energy X‐ray absorptiometry; EWGSOP, European Working Group on Sarcopenia in Older People; FNIH, Foundation for the National Institutes of Health; GS, gait speed; HGS, handgrip strength; ICS, International Consensus on Sarcopenia; SMI, skeletal muscle mass index.

### The overall prevalence of sarcopenia in patients with cardiovascular diseases and the general population

**FIGURE 2:**
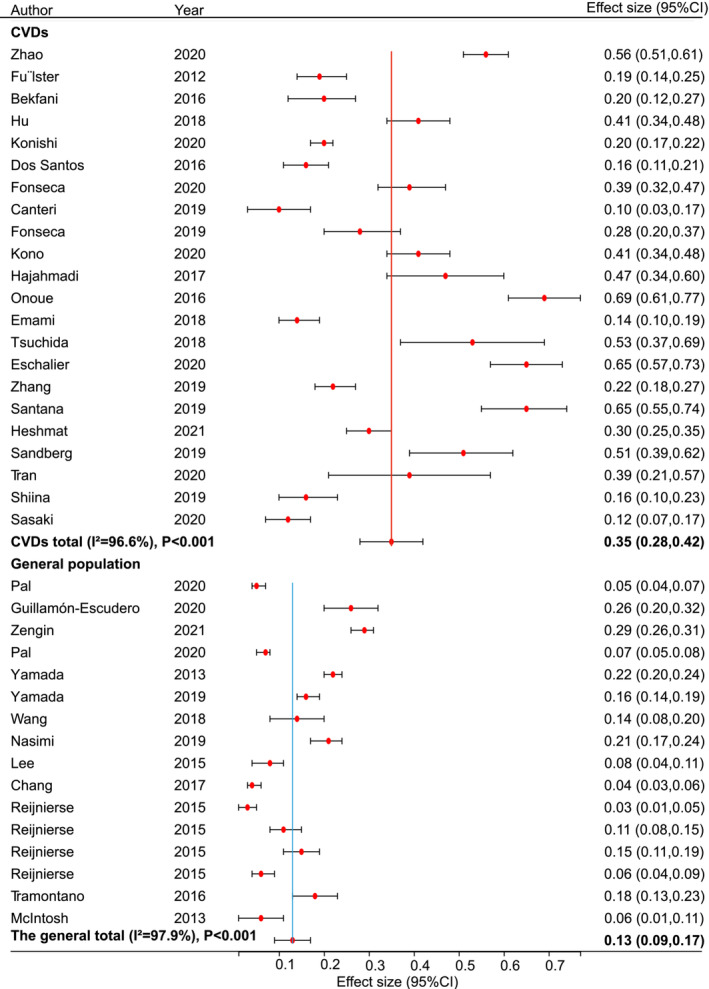
The pooled estimated prevalence rates of sarcopenia in patients with cardiovascular diseases (CVDs) and the general population. CI, confidence interval.

**FIGURE 3:**
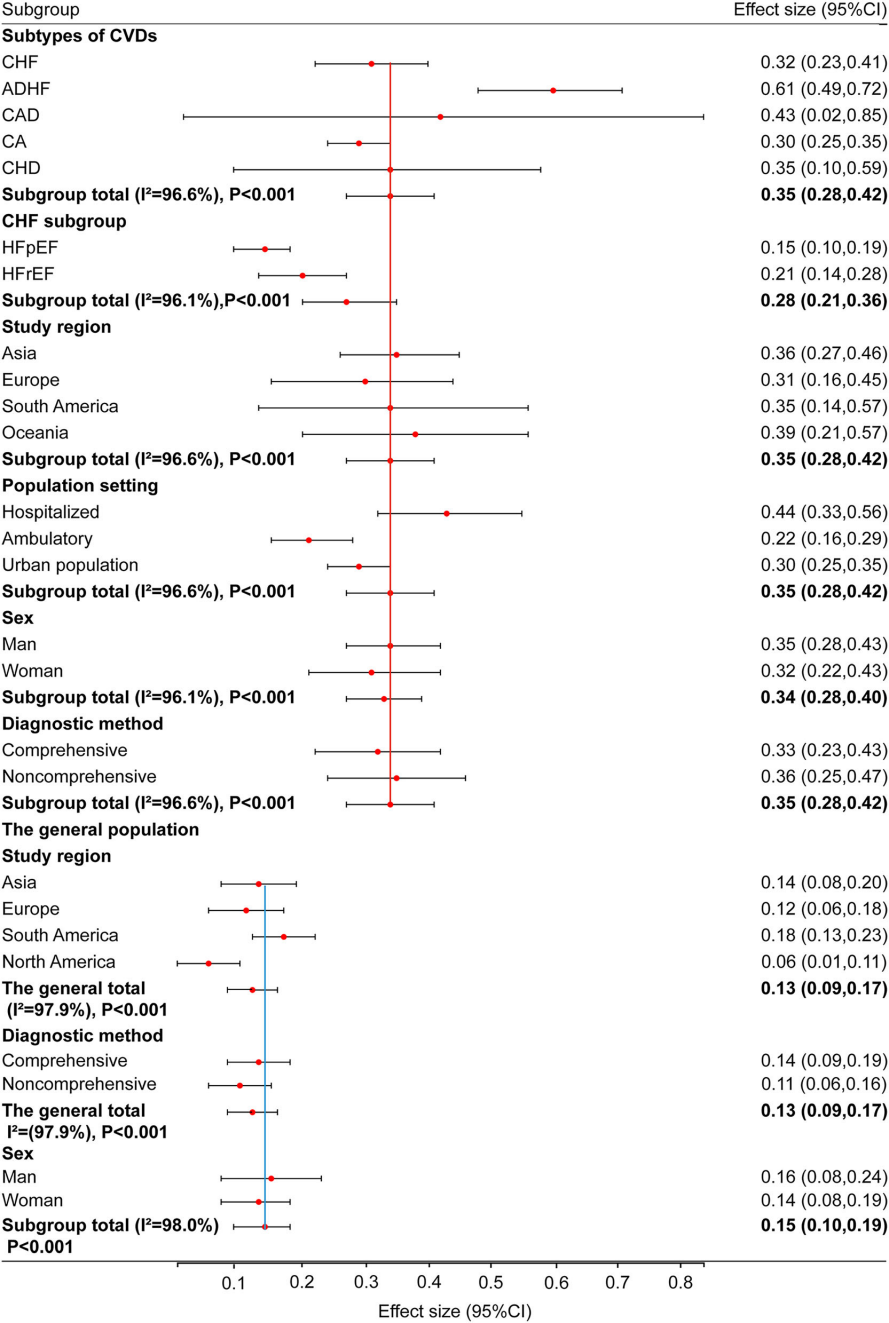
The pooled estimated prevalence rates of sarcopenia in patients with cardiovascular diseases (CVDs) and the general population by subgroups. ADHF, acute decompensated heart failure; CA, cardiac arrhythmia; CAD, coronary artery disease; CHD, congenital heart disease; CHF, chronic heart failure; CI, confidence interval; HFpEF, heart failure with preserved ejection fraction; HFrEF, heart failure with reduced ejection fraction.

In the 22 studies examining patients with CVDs, the prevalence of sarcopenia ranged widely from 10.1% to 68.9% (*Table* *1*), and the pooled prevalence was 35% (95% CI: 28–42%; *Figure* *2*). However, the pooled prevalence of sarcopenia in the general population (13%, 95% CI: 9–17%; *Figure* *2*) was significantly lower than that in patients with CVDs. Then, we stratified our analysis according to the subtypes of CVDs. The prevalence of sarcopenia in the general population was also remarkably lower than that in patients with acute decompensated heart failure (ADHF) (61%, 95% CI: 49–72%), chronic heart failure (CHF) (32%, 95% CI: 23–41%) and CA (30%, 95% CI: 25–35%), and the prevelance in the general was insignificantly compared with that in patients with CAD (43%, 95% CI: 2–85%) or CHD (35%, 95% CI: 10–59%; *Figure* *3*). Additionally, we stratified CHF into two categories based on ejection fraction, showing that the prevalence of sarcopenia in patients with heart failure with preserved ejection fraction (HFpEF) was 15% (95% CI: 10–19%) and that in patients with heart failure with reduced ejection fraction (HFrEF) was 21% (95% CI: 14–28%) (*Figure* *3*). However, the prevalence of sarcopenia in patients with HFpEF/HFrEF was not a significant difference from that in the general population.

### Subgroup analysis by study region, population setting, sex and sarcopenia diagnostic criteria

Our analysis was stratified according to four main study regions: Asia, Europe, South America and Oceania. The pooled prevalence of sarcopenia in patients with CVDs was 36% (95% CI: 27–46%) in Asia, 31% (95% CI: 16–45%) in Europe, 35% (95% CI: 14–57%) in South America and 39% (95% CI: 21–57%) in Oceania. The result revealed that there was no notable difference in the prevalence of sarcopenia among regions. In the included studies, the pooled prevalence of sarcopenia in hospitalized patients with CVDs was 44% (95% CI: 33–56%), that in ambulatory patients was 22% (95% CI: 16–29%) and that in the urban population was 30% (95% CI: 25–35%), exhibiting that the prevalence of sarcopenia in hospitalized patients was twice that in ambulatory patients. We also stratified our analysis according to sex. The pooled prevalence of sarcopenia was 35% (95% CI: 28–43%) in male patients with CVDs and 32% (95% CI: 22–43%) in female patients. For the general population, the prevalence of sarcopenia was 16% (95% CI: 8–24%) in males and 14% (95% CI: 8–19%) in females. For both patients with CVDs and the general population, there was no difference between males and females. Additionally, we stratified our analysis according to the method used to define sarcopenia in all 22 studies. The pooled prevalence of sarcopenia in patients with CVDs was 33% (95% CI: 23–43%) when using the comprehensive criteria and 36% (95% CI: 25–47%) when using the non‐comprehensive criteria, presenting again that different definitions of sarcopenia did not necessarily cause discrepancy in the prevalence of sarcopenia (*Figure* *3*).

### Publication bias and sensitivity analysis

There was significant publication bias detected in patients with CVDs as a whole (Egger's test: *P* = 0.012; Begg's test: *P* = 0.015; *Figure* *S1*), and the pooled measure was stable by the fill‐and‐trim method (*P* < 0.001; *Figure* *S2*). However, no publication bias was detected in patients with ADHF, CAD and CHD (Egger's test: *P* = 0.158; Begg's test: *P* = 1.000; *Figure* *S3*) or in patients with CHF, CA and unclassed CVDs (Egger's test: *P* = 0.092; Begg's test: *P* = 0.075; *Figure* *S4*). The finding revealed that the prevalence of sarcopenia in patients with total CVDs was discrepant. The results of the sensitivity analysis indicated that the stability of the prevalence of sarcopenia in patients with all CVDs was not influenced by a single study (*Figure* *S5*). In those relatively healthy populations, there was no publication bias (Egger's test: *P* = 0.256; Begg's test: *P* = 0.115; *Figure* *S6*) and the sensitivity analysis was robust (*Figure* *S7*).

## Discussion

### Summary of main results and mechanism basis

This systematic review would be the first comprehensive overview of the overall prevalence of sarcopenia in patients with different CVDs versus the prevalence of sarcopenia in the general population. The purpose of this systematic review was to investigate the latest evidence of the incidence of sarcopenia in patients with CVDs and in the general population and evaluate the correlation between sarcopenia and CVDs. The review showed that the prevalence rates of sarcopenia in patients with ADHF, CAD, CHD, CHF, CA and HFrEF were higher than those in the general population and that the prevalence of sarcopenia had statistical significance only in patients with ADHF, CHF and CA.

CVDs was characterized by a variety of pathological changes, such as systemic low‐level inflammation,^53^ neuroendocrine disorders,^28^ mitochondrial oxidative stress,^54^, ^55^ proteasome activation,^56^, ^57^ insulin resistance,^58^ endothelial dysfunction^28^ and low muscle blood flow.^28^ Among them, haemodynamic and metabolic changes were the two main culprits by which CVDs impair skeletal muscle.^59^ Low cardiac output led to the decrease of capillary density and oxygen diffusion in skeletal muscle,^28^, ^60^, ^61^ the conversion of skeletal muscle fibres from fast type II to slow type I and exercise intolerance.^7^, ^62^ Low‐grade persistent inflammation,^53^, ^63^ insulin resistance,^64^ sympathetic excitability^28^ and neuroendocrine disorders^65^ resulted in decreased skeletal muscle growth factor, overactivation of proteasome activity,^56^, ^57^ increased mitochondrial oxidative stress damage and the up‐regulation of reactive oxygen species,^56^, ^57^, ^66^ thereby inducing apoptosis^67^, ^68^ and leading to dysfunction of vascular endothelial cells and skeletal muscle stem cells.^28^, ^69^ These changes set off an imbalance between protein synthesis and protein degradation in skeletal muscle,^56^ which triggered the loss of skeletal muscle mass.^8^, ^16^ Hypercatabolism and activation of systemic immune responses in patients with HF may contribute to the attenuation of skeletal muscle mass.^22^, ^29^, ^70^ The failing heart caused lipolysis and skeletal muscle atrophy by secreting natriuretic peptide and soluble inhibins. However, the specific mechanism by which direct natriuretic peptides mediate the connection between failing heart and skeletal muscle had not been elucidated.^22^, ^29^, ^71^, ^72^ In the early stage of HF, ubiquitin‐proteasome system and autophagy were induced in skeletal muscle. Autophagy initiated through forkhead box O (FOXO) and AMP‐dependent protein kinase (AMPK) signalling under catabolic conditions leads to myofibril atrophy.^73^, ^74^ Additionally, the increased circulating sphingomyelinase activity and inflammatory factors (tumour necrosis factor α [TNFα] and soluble tumour necrosis factor receptor 1 [sTNF‐R1]) in HF patients can suppress sarcoplasmic reticulum Ca^2+^ release and blunt myofibrillar Ca^2+^ sensitivity, which can substantially reduce muscle strength and lean tissue mass.^75^, ^76^ This may be a possible mechanism contributing to disease‐related skeletal muscle dysfunction. Furthermore, gene set enrichment analysis showed that the proteasome degradation pathway was up‐regulated, whereas genes involved in muscle cell proliferation were down‐regulated.^77^ In turn, sarcopenia‐associated exercise intolerance, altered muscle metaboreflex and myokine dysregulation also contribute to the exacerbation of CVDs, forming a vicious circle.

Inadequate exercise was a common risk factor for CVDs and sarcopenia. Physical training was recognized as an effective nonpharmacological treatment strategy for a healthy lifestyle and for CVDs and sarcopenia.^78^, ^79^ Exercise increased cardiac output, better delivery of oxygen to peripheral tissues and improved cardiopulmonary health and skeletal muscle functional adaptations.^80^, ^81^ Mounting evidence showed that resistance exercise (RE) improved skeletal muscle strength, muscle quality and mass, and physical function in middle‐aged and older adults.^82^, ^83^, ^84^, ^85^, ^86^ RE increased microvascular blood volume and cross‐sectional area of type I and II fibres, especially type II fibre, which were the central characteristics of sarcopenia.^84^, ^87^ Compared with low‐intensity RE, moderate‐ and high‐intensity resistance training significantly enhanced skeletal muscle perfusion and muscle strength and mass. Appropriate prescription of RE served a promising and crucial treatment for sarcopenia.^79^ Mechanistically, exercise promotes the Ca^2+^ cycling and ATP turnover in skeletal muscle.^88^ Binding of AMP to the heterotrimeric AMPK activated AMPK and its downstream peroxisome proliferator‐activated receptor‐γ coactivator‐1α (PGC‐1α) phosphorylation, ultimately leading to increased fatty acid oxidation and glucose uptake, increased mitochondrial content and function, and degradation of dysfunctional mitochondrial fragments by mitochondrial phagocytosis.^89^, ^90^, ^91^, ^92^ During acute elevations in muscle metabolism, AMPK had been localized as a key regulator of mitochondrial biogenesis and degradation.^93^ Meantime, some researchers demonstrated that muscle mass and muscle strength have a protective effect on HF patients.^94^, ^95^ Therefore, it is important to screen high‐risk groups of sarcopenia or pre‐sarcopenia in order to do an early intervention, especially exercise, to reverse or slow down the occurrence and development of sarcopenia.

### Subgroup analysis of different cardiovascular disease types

In the meta‐analysis, we conducted a subgroup analysis and concluded that sarcopenia prevalence in patients with different subtypes of CVDs was higher than that in the general population. The prevalence of sarcopenia was higher in patients with ADHF, CHF and CA than that in the general population, which was a statistically significant result. We inferred that sarcopenia might be the cause of acute onset and exacerbation in ADHF and CA, whereas a positive relationship of comorbidities might exist between CHF and sarcopenia, which may explain that the sarcopenia group had higher brain natriuretic peptide levels than the non‐sarcopenia group.^28^, ^29^ This finding could be explained by two major factors. First, patients with ADHF and CA might have concurrent muscle loss before admission due to haemodynamic changes and may aggravate or worsen ADHF and CA symptoms due to the different severity and duration of disease, which further affect body composition and skeletal muscle function. Second, HF patients were generally accompanied by complications such as oedema, decreased physical activity and malnutrition, resulting in selection bias and measurement error in the evaluation of sarcopenia. It is undeniable that only two eligible ADHF studies met the inclusion criteria, and both had a higher prevalence rate than the pooled rate. Although the authenticity and effectiveness of high prevalence were verified in one study, the limited sample size might be the reason for the heterogeneity in another study. The only CA study demonstrated and emphasized that sarcopenia, especially LMS or low muscle performance, was closely related to severe arrhythmia in the elderly.^12^ Meanwhile, sarcopenia accounted for 22.2% of minor electrocardiograph abnormalities. In previous studies, sarcopenia was associated with atrial fibrillation, congestive HF and CAD.^14^, ^15^ The sample size of this study was large, but it did not cover all continents and all populations. The incidence of sarcopenia in patients with CHF in this study was higher than that of previous studies^6^, ^7^, ^21^, ^33^ and ranged from 10.1% to 68.9%. A prospective multinational Studies Investigating Co‐morbidities Aggravating Heart Failure (SICA‐HF) study showed that sarcopenia was a concomitant symptom in patients with CHF.^6^ Sarcopenia was defined as an important comorbidity of HF, which required special attention, in the 2016 HF guidelines of the European Society of Cardiology.^96^ It had been demonstrated that skeletal muscle mass (including fat‐free mass and appendicular lean mass) improved significantly during the first 6 months of left ventricular assist device support in patients accompanied by advanced systolic HF and high prevalence of muscle loss.^97^

We performed an analysis of CHF and found that the pooled prevalence rates of sarcopenia in patients with HFpEF and HFrEF were insignificant and consistent with previous studies.^22^, ^31^ The incidence of sarcopenia in patients with HFrEF was higher than that in the general population. Undeniably, we had more HFrEF studies than HFpEF studies, and the sample size of HFrEF was greater than that of HFpEF. Due to differences in left ventricular ejection fraction (LVEF) and exercise capacity between HFpEF and HFrEF, their impacts on sarcopenia are different. In recent years, with the aging of the population, HFpEF has become the main form of global HF (>50%).^98^ Compared with HFrEF, the incidence rate of HFpEF increased by 10% every 10 years, and the gap might continue to widen.^99^, ^100^ Skeletal muscle atrophy, fat infiltration and exercise intolerance lead to myocardial hypertrophy and diastolic dysfunction. Additionally, previous studies showed that the risk of diastolic dysfunction in sarcopenia patients was greater than that in non‐sarcopenia patients.^101^, ^102^ In other words, sarcopenia was more likely to develop in HFpEF patients. Meanwhile, sarcopenia with obesity further increased the risk of diastolic dysfunction.^103^ Because HFpEF patients have exercise intolerance and the current medical treatment lacks effective intervention for such patients, ameliorating sarcopenia and obesity may be the potential treatment targets of HFpEF patients. The decrease of LVEF might result in low blood flow change of skeletal muscle and low‐grade inflammation of HFrEF, increasing sarcopenia prevalence. Sarcopenia was an independent predictor of 1‐year mortality and contributed to mortality similarly in HFpEF and HFrEF.^21^

Additionally, although the prevalence of sarcopenia was higher in patients with CHD and CAD than that in the general population, there were no statistical differences. Thus, more prospective multicentre studies are needed for further validation. Sarcopenia is commonly seen in older adults, but studies show that it also affects younger generations, for example, young adults with CHD. Compared with non‐diseased controls, adults with complex CHD had a higher prevalence of sarcopenia by the LMM and LMS diagnosis criteria.^26^ Therefore, considering susceptibility to sarcopenia, the use of the LMM diagnosis criteria alone for sarcopenia may result in a higher prevalence than the adoption of the comprehensive criteria. In addition, for CHD patients, the skeletal muscle mass index (SMI) and BMI in the sarcopenia group were lower and the oedema index was higher compared with the non‐sarcopenia group.^10^ Skeletal muscle wasting, growth deficit and hypoplasia in patients with Fontan circulation jointly led to secondary muscle loss.^11^ Accumulative skeletal muscle damage triggered the onset of CHD and CAD due to irregular exercise and endothelial dysfunction. In addition, Sasaki et al.^23^ found that patients with CVDs had a lower motor function and a higher prevalence of sarcopenia compared with community participants. Nevertheless, Shiina et al.^26^ demonstrated that appropriate nutritional supplementation and resistance training (amino acid intake plus resistance training) actively improved skeletal muscle mass, body fat percentage and oedema in patients with CHD. It is reported that high‐intensity training (HIT), including aerobic interval training, could increase cardiac output of patients with CVDs and improve cardiovascular system and skeletal muscle perfusion.^80^, ^81^, ^104^ HIT might improve the risk factors of cardiovascular metabolism, such as improving blood pressure, BMI and increasing insulin sensitivity. It mainly drove mitochondrial biogenesis, sarcoplasmic reticulum calcium uptake and glucose transporter adaptation through the increase of PGC‐1α.^80^, ^105^, ^106^ Thus, regular resistance and aerobic exercise improved cardiovascular and skeletal muscle health, offset or significantly delayed the progress of muscle atrophy. Limitations in the number of studies, sample size and differences in the diagnosis of sarcopenia and subtypes of CVDs led to a higher incidence of sarcopenia in patients with CVDs than the general population and heterogeneity between groups.

### Subgroup analysis of study region, sex, diagnosis criteria of sarcopenia and population setting

There was no statistical difference between subgroups when the analysis was stratified by study region, sex and diagnosis criteria of sarcopenia. Although the prevalence was highest in Oceania, unfortunately, only one study met the criteria. We found that the prevalence of sarcopenia in women and men is almost identical, which is consistent with previous studies.^37^, ^69^ The prevalence of sarcopenia in studies using non‐comprehensive methods seemed to be higher than in those using comprehensive methods (36% vs. 33%), but this difference was not statistically significant considering different criteria used to define sarcopenia and specified cut‐off points. At present, there is no clear and widely applicable definition of measurement standards for sarcopenia around the world.^107^ On the other hand, there is considerable room for improvement in the methodological quality of clinical diagnosis for sarcopenia in the international clinical practice guidelines of sarcopenia.^108^ The prevalence of sarcopenia in hospitalized patients was significantly higher than that in outpatients and the urban population. This meant that hospitalized patients had more severe CVDs than the others. Of all the included studies, the number of hospitalized studies was greater than that of outpatient studies, which indicated that more sarcopenia cases were found in hospitals and that regular screening for sarcopenia was necessary.

### Reasons for publication bias

Most of the included studies were of high quality and with low risk of bias. Although the publication bias was significant in patients with CVDs as a whole, no publication bias was detected in any subtype of CVDs. On the one hand, sarcopenia is an inducement of acute onset or aggravation of ADHF, CAD, CA and CHD. On the other, sarcopenia is a comorbidity of CHF, and their long‐term interaction leads to the high incidence of sarcopenia in patients with CHF. That explains the disparities in the prevalence rates of sarcopenia in patients with different CVDs.

### Reasons for the heterogeneity

Overall, intergroup heterogeneity could be attributed to four factors. First, there are different diagnostic methods and inclusion/exclusion criteria for subtypes of CVDs. Second, CVDs with special physiological and pathological mechanisms have different effects on skeletal muscle, thus influencing the incidence of sarcopenia to varying degrees. Third, the study regions, population backgrounds and gender composition jointly affected the comorbidity rate. Finally, the limitations of study number and sample size should be considered.

### Strengths and limitations

In the current meta‐analysis, we considered our literature search comprehensive without omitting any relevant trials. We used a standard scale to evaluate the quality of the included literature and repeatedly validated the meta‐results. Although the results were encouraging, the study had several limitations. First, the research on sarcopenia related to CVDs and their subtypes was insufficient, and large sample and multicentre studies were needed to prove the causality. In addition, subtypes of sarcopenia and the severity of CVDs were not analysed. Second, the cross‐sectional and small cohort designs limited the possibilities of determining the causal relationship between sarcopenia and CVDs. Third, there was significant heterogeneity among the included studies when stratified by subtype of CVDs, population setting, diagnostic methods, sex and study region. Moreover, the prevalence of sarcopenia in patients with CVDs only represented the results of univariate analysis, whereas confounding factors, such as CVDs drugs, New York Heart Association functional classification, LVEF and the number of complications, could affect skeletal muscle, but these factors were not available and had not been analysed. Additionally, the study only focused on the research population of four continents, excluding the research in North America, which limited its global applicability.

## Conclusions

Sarcopenia is a common disease with aging. Our results showed that the prevalence of sarcopenia in patients with CVDs was higher than that in the general population, especially in patients with ADHF, CA and CHF, suggesting that there is a positive correlation between CVDs and sarcopenia. Patients with CVDs might be accompanied by sarcopenia due to the common aetiology and pathogenesis, and their survival rate and prognosis were poor. In clinical practice, early screening and physical interventions of high‐risk or probable sarcopenia in patients with CVDs can improve the prognosis and quality of life and further reduce the social and economic burden of the CVDs population. The prevalence of sarcopenia will likely increase as the population continues to age. Future research should focus on identifying the causality link between CVDs and sarcopenia in terms of common risks and potential mechanisms, so as to fight sarcopenia in a more effective and precise way.

## Funding

This study was supported in part by grants from the National Key Research and Development Program of China (2020YFC2005601), the Key Research Program of National Clinical Research Center for Geriatrics (Z20191004), the National Natural Science Foundation of China (81970715) and the Innovation Spark Project of Sichuan University (2018SCUH0065).

## Conflict of interest statement

None declared.

## Supporting information


**Table S1.** Search strategy by Pubmed, Embase, Medline, and Web of Science by Ovid SP
**Table S2.** Characteristics of the included studies and main outcome in the general population
**Table S3.** Risk of bias of the included studies using the National Institutes of Health Quality Assessment Tool for Observational Cohort and Cross‐Sectional Studies
**Table S4.** Risk of bias of the included studies using assessment tool explicitly for prevalence studies
**Table S5.** The details of diagnostic criteria and cut‐off points of each study in general populations
**Figure S1.** The Egger's funnel plot of total CVDs in the meta‐analysis using Egger's test
**Figure S2.** The Egger's funnel plot of total CVDs by the fill‐and‐trim method
**Figure S3.** The Egger's funnel plot of ADHF, CAD and CHD studies in the meta‐analysis using Egger's test
**Figure S4.** The Egger's funnel plot of CHF, CA and unclassed CVDs studies in the meta‐analysis using Egger's test
**Figure S5.** Sensitivity analysis for the effect of individual studies (given named study in the Y axis is omitted) on the pooled prevalence of sarcopenia in CVDs. CI, confidence interval
**Figure S6.** The Egger's funnel plot of general population in the meta‐analysis using Egger's test
**Figure S7.** Sensitivity analysis for the effect of individual studies (given named study in the Y axis is omitted) on the pooled prevalence of sarcopenia in general populations. CI, confidence interval
