# Supplementary material for: Sarcopenia and cardiovascular diseases: A systematic review and meta‐analysis
Source: J Cachexia Sarcopenia Muscle. 2023 Apr 1;14(3):1183–98. doi: 10.1002/jcsm.13221 (PMC10235887; doi:10.1002/jcsm.13221)
Supplement: Supplementary file 1 — Table S1. Search strategy by Pubmed, Embase, Medline, and Web of Science by Ovid SP Table S2. Characteristics of the included studies and main outcome in the general population Table S3. Risk of bias of the included studies using the National Institutes of Health Quality Assessment Tool for Observational Cohort and Cross‐Sectional Studies Table S4. Risk of bias of the included studies using assessment tool explicitly for prevalence studies Table S5. The details of diagnostic criteria and cut‐off points of each study in general populations Figure S1. The Egger's funnel plot of total CVDs in the meta‐analysis using Egger's test Figure S2. The Egger's funnel plot of total CVDs by the fill‐and‐trim method Figure S3. The Egger's funnel plot of ADHF, CAD and CHD studies in the meta‐analysis using Egger's test Figure S4. The Egger's funnel plot of CHF, CA and unclassed CVDs studies in the meta‐analysis using Egger's test Figure S5. Sensitivity analysis for the effect of individual studies (given named study in the Y axis is omitted) on the pooled prevalence of sarcopenia in CVDs. CI, confidence interval Figure S6. The Egger's funnel plot of general population in the meta‐analysis using Egger's test Figure S7. Sensitivity analysis for the effect of individual studies (given named study in the Y axis is omitted) on the pooled prevalence of sarcopenia in general populations. CI, confidence interval [file JCSM-14-1183-s001.docx]

**Sarcopenia and Cardiovascular Diseases: A Systematic Review and Meta-analysis**

**Table S1** Search strategy by Pubmed, Embase, Medline, and Web of Science by Ovid SP

1 exp sarcopenia/

2 (sarcopeni$ or myopeni$ or dynaponi$).tw.

3 (muscle atroph$ or muscle wasting$ or muscle weak$ or muscle loss$ or muscular atroph$ or muscular wasting$ or muscular weak$ or muscular loss$).tw.

4 1 or 2 or 3

5 exp coronary artery disease/

6 (Coronary Diseases or Disease, Coronary or Diseases, Coronary or Coronary Heart Disease or Coronary Heart Diseases or Disease, Coronary Heart or Diseases, Coronary Heart or Heart Disease, Coronary or Heart Diseases, Coronary or CAD or CHD).ab,kw.

7 (Artery Disease, Coronary or Artery Diseases, Coronary or Coronary Artery Diseases or Left Main Coronary Artery Disease or Left Main Disease or Left Main Diseases or Left Main Coronary Disease or Coronary Arteriosclerosis or Arterioscleroses, Coronary or Coronary Arterioscleroses or Atherosclerosis, Coronary or Atheroscleroses, Coronary or Coronary Atheroscleroses or Coronary Atherosclerosis or Arteriosclerosis, Coronary).ab,kw.

8 5 or 6 or 7

9 4 and 8

10 exp heart failure/

11 (Cardiac Failure or Heart Decompensation or Decompensation, Heart or Heart Failure, Right-Sided or Heart Failure, Right Sided or Right-Sided Heart Failure or Right Sided Heart Failure or Myocardial Failure or Congestive Heart Failure or Heart Failure, Congestive or Heart Failure, Left-Sided or Heart Failure, Left Sided or Left-Sided Heart Failure or Left Sided Heart Failure or HF or CHF).ab,kw.

12 10 or 11

13 4 and 12

14 exp heart muscle ischemia/

15 (Ischemia$, Myocardial or Myocardial Ischemia$ or Ischemic Heart Disease$ or Heart Disease$, Ischemic or Disease$, Ischemic Heart).ab,kw.

16 14 or 15

17 4 and 16

18 exp cardiomyopathy/

19 (cardiomyopathy or Myocardiopath$ or Myocardial Disease$ or Disease$, Myocardial or Cardiomyopath$, Secondary or Secondary Cardiomyopath$ or Secondary Myocardial Disease$ or Myocardial Disease$, Secondary or Disease$, Secondary Myocardial or Cardiomyopath$, Primary or Primary Cardiomyopath$ or Primary Myocardial Disease$ or Myocardial Disease$, Primary or Disease$, Primary Myocardial).ab,kw.

20 18 or 19

21 4 and 20

22 exp valvular heart disease/

23 (Heart Valve Disease$ or Valve Disease$, Heart or Heart Valvular Disease$ or Disease$, Heart Valvular or Valvular Heart Disease$).ab,kw.

24 22 or 23

25 4 and 24

26 congenital heart malformation/

27 (Defect$, Congenital Heart or Abnormalit$, Heart or Heart Abnormalit$ or Congenital Heart Defect$ or Malformation Of Heart$ or Heart Defect$, Congenital or Congenital Heart Disease$ or Disease$, Congenital Heart or Heart Disease$, Congenital).ab,kw.

28 26 or 27

29 4 and 28

30 exp cardiovascular disease/

31 (Cardiovascular Disease$ or Disease$, Cardiovascular).ab,kw.

32 30 or 31

33 4 and 32

34 exp prevalence ratio/

35 exp prevalence/

36 exp morbidity/

37 exp incidence/

38 (prevalence or prevalence rate or morbidity or incidence or incidence rate).ab,kw.

39 34 or 35 or 36 or 37 or 38

40 4 and 39

**Table S2** Characteristics of the included studies and main outcome in the general population

| First author and year | Country | Study region | Study design | population setting | Sample size | Male, n(%) | Female, n(%) | Age  (years)^a^ | Prevalence of sarcopenia, n(%) | | | Criteria (assessment method to detect sarcopenia) | HGS measure hand | Cut-off value for HGS (kg) | Sarcopenia diagnostic criteria |
| --- | --- | --- | --- | --- | --- | --- | --- | --- | --- | --- | --- | --- | --- | --- | --- |
|  |  |  |  |  |  |  |  |  | total, n(%) | male, n(%) | female, n(%) |  |  |  |  |
| Pal^1^ (2020) | India | Asia | cross-sectional | community | 804 | 339  (42.2) | 465  (57.8) | 44.4 | 42  (5.2) | 35  (10.3) | 7  (1.5) | LMS+LMM | dominant | HGS < 27 kg (males)/16 kg (females) | EWGSOP  2018 |
| Pal^1^ (2020) | India | Asia | cross-sectional | community | 804 | 339  (42.2) | 465  (57.8) | 44.4 | 54  (6.7) | 29  (8.5) | 25  (5.3) | LMS+LMM | dominant | HGS < 28 kg (males)/18 kg (females) | AWGS  2019 |
| Guillamón-Escudero^2^ (2020) | Spain | Europe | cross-sectional | community | 202 | 38  (18.8) | 164  (81.2) | 73.01 | 53  (26.2) | 11  (28.9) | 42  (25.6) | LMS+LMM and /or LPP | each/chair stand | HGS < 27 kg (males)/16 kg (females)/Chair stand >15 s for five rises | EWGSOP  2018 |
| Zengin^3^  (2021) | India | Asia | cohort | community | 1754 | 1013  (57.8) | 741  (42.2) | 52.2 | 501  (28.6) | 355  (35.0) | 146  (19.7) | LMS+LMM | each | HGS < 27 kg (males)/16 kg (females) | EWGSOP  2018 |
| Yamada (2013) | japan | Asia | cross-sectional | community | 1882 | 568 (30.2) | 1314 (69.8) | 74.9 | 414 (22.0) | 124 (21.8) | 290 (22.1) | LMS+LMM+LPP | dominant | HGS < 30 kg (males)/20 kg (females) | EWGSOP  2010 |
| Yamada (2019) | japan | Asia | cross-sectional | community | 1009 | 285 (28.2) | 724 (71.8) | 80.6 | 166 (16.5) | 61 (21.4) | 105 (14.5) | LMS+LMM and /or LPP | dominant | HGS < 26 kg (males)/18 kg (females) | AWGS 2014 |
| Wang (2018) | China | Asia | cross-sectional | community | 135 | 39 (28.9) | 96 (71.1) | 71.2 | 19 (14.0) | NA | NA | LMM | both hands | HGS < 26 kg (males)/18 kg (females) | AWGS 2014 |
| Nasimi (2019) | Iran | Asia | cross-sectional | community | 501 | 254 (50.7) | 247 (49.3) | 70.3 | 104 (20.8) | 70 (27.6) | 34 (13.8) | LMS+LMM and/or LPP | both hands | HGS < 26 kg (males)/18 kg (females) | AWGS 2014 |
| Lee (2015) | Korea | Asia | cross-sectional | ambulatory | 196 | 0 (0.0) | 196 (100) | 71.2 | 15 (7.6) | 0.0 | 15 (7.6) | LMS+LMM and/or LPP | dominant | HGS < 18 kg (females) | AWGS 2014 |
| Chang (2017) | Korea | Asia | cross-sectional | community | 715 | 325 (45.5) | 390 (54.5) | 66.5 | 31 (4.3) | 12 (3.7) | 19 (4.9) | LMS+LMM | both hands | HGS < 26 kg (males)/18 kg (females) | AWGS 2014 |
| Reijnierse (2015) | UK, France, Netherlands, Estonia and Finland | Europe | cross-sectional | community | 308 | 152 (49.4) | 156 (50.6) | 74.4 | 20 (6.5) | NA | NA | LMS+LMM | dominant | HGS < 30 kg (males)/20 kg (females) | EWGSOP  2010 |
| Reijnierse (2015) | UK, France, Netherlands, Estonia and Finland | Europe | cross-sectional | community | 308 | 152 (49.4) | 156 (50.6) | 74.4 | 20 (6.5) | NA | NA | LMM | NA | NA | EWGSOP  2010 |
| Reijnierse (2015) | UK, France, Netherlands, Estonia and Finland | Europe | cross-sectional | community | 308 | 152 (49.4) | 156 (50.6) | 74.4 | 20 (6.5) | NA | NA | LMM | NA | NA | EWGSOP  2010 |
| Reijnierse (2015) | UK, France, Netherlands, Estonia and Finland | Europe | cross-sectional | community | 308 | 152 (49.4) | 156 (50.6) | 74.4 | 20 (6.5) | NA | NA | LMS | both hands | HGS < 30.3 kg (males)/19.3 kg (females) | Others^b^ |
| Tramontano (2016) | Peru | South America | cross-sectional | rural | 222 | 102 (45.9) | 120 (50.1) | 74 | 39 (16.7) | 1 (1.0) | 38 (31.7) | LMM+LPP | NA | NA | IWGS 2011 |
| McIntosh (2013) | Canada | North America | cross-sectional | community | 85 | 42 (49.4) | 43 (50.6) | 7.2 | 5 (6.0) | 2 (5.0) | 3 (7.0) | LMS+LMM and /or LPP | both hands | HGS < 30 kg (males)/20 kg (females) | EWGSOP 2010 |

Abbreviations: ASM: appendicular lean mass; ASMI: appendicular lean mass index; AWGS, Asian Working Group for Sarcopenia; BIA, bioelectrical impedance analysis; DXA, dual-energy X-ray absorptiometry; EWGSOP, European Working Group on Sarcopenia in Older People; GS: gait speed; HGS: handgrip strength; LMM, lower muscle mass; LMS, lower muscle strength; LPP, lower physical performance; NA, not applicable; NR, not reported; SPPB: short physical performance battery; 5CST: 5-time chair stand test.

^a^Mean or median as reported.

^b^Sarcopenia diagnostic criteria other than EWGSOP (2010), EWGSOP2 (2018), AWGS (2014), AWGS (2019), FNIH and IWGS (2011).

**Table S3** Risk of bias of the included studies using the National Institutes of Health Quality Assessment Tool for Observational Cohort and Cross-Sectional Studies

| First author(year) | Q1 | Q2 | Q3 | Q4 | Q5 | Q6 | Q7 | Q8 | Q9 | Q10 | Q11 | Q12 | Q13 | Q14 |
| --- | --- | --- | --- | --- | --- | --- | --- | --- | --- | --- | --- | --- | --- | --- |
| Zhao (2020) | Yes | Yes | Yes | Yes | NA | No | No | NA | Yes | NA | Yes | NA | NA | NA |
| Fu¨lster (2012) | Yes | Yes | Yes | Yes | Yes | No | No | NA | Yes | NA | Yes | NA | NA | Yes |
| Bekfani (2016) | Yes | Yes | Yes | Yes | Yes | No | No | NA | Yes | NA | Yes | NA | NA | Yes |
| Hu (2018) | Yes | Yes | Yes | Yes | Yes | No | No | NA | Yes | NA | Yes | NA | Yes | NA |
| Konishi (2020) | Yes | Yes | Yes | Yes | Yes | Yes | Yes | NA | Yes | NA | Yes | NA | Yes | Yes |
| Tsuchida (2018) | Yes | Yes | No | Yes | No | Yes | Yes | NA | Yes | NA | Yes | NA | NA | No |
| Eschalier (2020) | Yes | Yes | Yes | Yes | Yes | Yes | Yes | NA | Yes | Yes | Yes | NA | NA | No |
| Dos Santos (2016) | Yes | Yes | Yes | Yes | No | No | No | NA | Yes | Yes | Yes | NA | NA | No |
| Fonseca (2020) | Yes | Yes | Yes | Yes | No | No | No | NA | Yes | NA | Yes | NA | NA | NA |
| Canteri (2019) | Yes | Yes | No | Yes | Yes | No | No | NA | Yes | NA | Yes | NA | NA | No |
| Fonseca (2019) | Yes | Yes | Yes | Yes | No | No | No | NA | Yes | NA | Yes | NA | NA | NA |
| Kono (2020) | Yes | Yes | Yes | Yes | No | No | No | NA | Yes | NA | Yes | NA | NA | NA |
| Hajahmadi (2017) | Yes | Yes | Yes | Yes | Yes | No | No | NA | Yes | NA | Yes | NA | NA | NA |
| Onoue (2016) | Yes | Yes | Yes | Yes | Yes | Yes | Yes | NA | Yes | NA | Yes | NA | Yes | Yes |
| Emami (2018) | Yes | Yes | Yes | Yes | Yes | No | No | NA | Yes | NA | Yes | NA | NA | NA |
| Zhang (2019) | Yes | Yes | Yes | Yes | Yes | No | No | NA | Yes | NA | Yes | NA | NA | NR |
| Santana (2019) | Yes | Yes | Yes | Yes | Yes | No | No | NA | Yes | NA | Yes | NA | NA | NA |
| Heshmat (2021) | Yes | Yes | Yes | Yes | Yes | No | No | NA | Yes | NA | Yes | NA | NA | Yes |
| Sandberg (2019) | Yes | Yes | Yes | Yes | Yes | No | No | NA | Yes | NA | Yes | NA | NA | NA |
| Tran (2020) | Yes | Yes | Yes | Yes | Yes | No | No | NA | Yes | NA | Yes | NA | NA | NA |
| Shiina (2019) | Yes | Yes | Yes | Yes | Yes | No | No | NA | Yes | NA | NA | NA | NA | NA |
| Sasaki (2020) | Yes | Yes | Yes | Yes | Yes | No | No | NA | Yes | NA | Yes | NA | NA | NA |
| Pal (2020) | Yes | Yes | Yes | Yes | Yes | Yes | NA | NA | Yes | NA | Yes | NA | NA | NA |
| Yamada (2013) | Yes | Yes | Yes | Yes | No | Yes | No | NA | Yes | NA | Yes | NA | NA | No |
| Yamada (2019) | Yes | Yes | Yes | Yes | No | Yes | No | NA | Yes | NA | Yes | NA | NA | No |
| Guillamón-Escudero (2020) | Yes | Yes | Yes | Yes | Yes | Yes | NA | NA | Yes | NA | Yes | NA | NA | NA |
| Wang (2018) | Yes | Yes | Yes | Yes | No | Yes | No | NA | Yes | NA | Yes | NA | NA | NA |
| Nasimi (2019) | Yes | Yes | Yes | Yes | No | Yes | No | NA | Yes | NA | Yes | NA | NA | NA |
| Lee (2015) | Yes | Yes | Yes | Yes | No | Yes | No | NA | Yes | NA | Yes | NA | NA | NA |
| Zengin (2021) | Yes | Yes | Yes | Yes | No | Yes | NA | NA | Yes | NA | Yes | NA | NA | NA |
| Chang (2021) | Yes | Yes | Yes | Yes | No | Yes | NA | NA | Yes | NA | Yes | NA | NA | Yes |
| Reijnierse (2015) | Yes | Yes | Yes | Yes | No | Yes | NA | NA | Yes | NA | Yes | NA | NA | NA |
| Tramontano (2016) | Yes | Yes | Yes | Yes | No | Yes | NA | NA | Yes | NA | Yes | NA | NA | Yes |
| McIntosh (2013) | Yes | Yes | Yes | Yes | No | Yes | NA | NA | Yes | NA | Yes | NA | NA | NA |

Yes = criteria met No = criteria not met NA = not applicable NR = not reported

Criteria

Q1. Was the research question or objective in this paper clearly stated?

Q2. Was the study population clearly specified and defined?

Q3. Was the participation rate of eligible persons at least 50%?

Q4. Were all the subjects selected or recruited from the same or similar populations (including the same time period)? Were inclusion and exclusion criteria for being in the study prespecified and applied uniformly to all participants?

Q5. Was a sample size justification, power description, or variance and effect estimates provided?

Q6. For the analyses in this paper, were the exposure(s) of interest measured prior to the outcome(s) being measured?

Q7. Was the timeframe sufficient so that one could reasonably expect to see an association between exposure and outcome if it existed?

Q8. For exposures that can vary in amount or level, did the study examine different levels of the exposure as related to the outcome (e.g., categories of exposure, or exposure measured as continuous variable)?

Q9. Were the exposure measures (independent variables) clearly defined, valid, reliable, and implemented consistently across all study participants?

Q10. Was the exposure(s) assessed more than once over time?

Q11. Were the outcome measures (dependent variables) clearly defined, valid, reliable, and implemented consistently across all study participants?

Q12. Were the outcome assessors blinded to the exposure status of participants?

Q13. Was loss to follow-up after baseline 20% or less?

Q14. Were key potential confounding variables measured and adjusted statistically for their impact on the relationship between exposure(s) and outcome(s)?

**Table S4** Risk of bias of the included studies using assessment tool explicitly for prevalence studies

| First author(year) | Q1 | Q2 | Q3 | Q4 | Q5 | Q6 | Q7 | Q8 | Q9 | Q10 | Overall |
| --- | --- | --- | --- | --- | --- | --- | --- | --- | --- | --- | --- |
| Zhao (2020) | High | Low | High | Low | Low | Low | Low | Low | Low | Low | Low |
| Fu¨lster (2012) | High | Low | High | Low | Low | Low | Low | Low | Low | Low | Low |
| Bekfani (2016) | High | Low | High | Low | Low | Low | Low | Low | Low | Low | Low |
| Hu (2018) | High | Low | High | Low | Low | Low | Low | Low | Low | Low | Low |
| Konishi (2020) | High | Low | High | High | Low | Low | Low | Low | Low | Low | Moderate |
| Tsuchida (2018) | High | Low | High | High | Low | Low | Low | Low | Low | Low | Moderate |
| Eschalier (2020) | High | Low | High | Low | Low | Low | Low | Low | Low | Low | Low |
| Dos Santos (2016) | High | Low | High | Low | Low | Low | Low | Low | Low | Low | Low |
| Fonseca (2020) | High | Low | High | Low | Low | Low | Low | Low | Low | Low | Low |
| Canteri (2019) | High | Low | High | Low | Low | Low | Low | Low | Low | Low | Low |
| Fonseca (2019) | High | Low | High | Low | Low | Low | Low | Low | Low | Low | Low |
| Kono (2020) | High | Low | High | Low | Low | Low | Low | Low | Low | Low | Low |
| Hajahmadi (2017) | High | Low | High | Low | Low | Low | Low | Low | Low | Low | Low |
| Onoue (2016) | High | Low | High | Low | Low | Low | Low | Low | Low | Low | Low |
| Emami (2018) | High | Low | High | Low | Low | Low | Low | Low | Low | Low | Low |
| Zhang (2019) | High | Low | High | Low | Low | Low | Low | Low | Low | Low | Low |
| Santana (2019) | High | Low | High | High | Low | Low | Low | Low | Low | Low | Moderate |
| Heshmat (2021) | Moderate | Low | Low | Low | Low | Low | Low | Low | Low | Low | Low |
| Sandberg (2019) | High | Low | High | Low | Low | Low | Low | Low | Low | Low | Low |
| Tran (2020) | High | Low | High | Low | Low | Low | Low | Low | Low | Low | Low |
| Shiina (2019) | High | Low | High | High | Low | Low | Low | Low | Low | Low | Moderate |
| Sasaki (2020) | High | Low | High | Low | Low | Low | Low | Low | Low | Low | Low |
| Pal (2020) | High | Low | Low | Low | Low | Low | Low | Low | Low | Low | Low |
| Yamada (2019) | High | Low | High | High | Low | Low | Low | Low | Low | Low | Moderate |
| Yamada (2013) | High | Low | High | High | Low | Low | Low | Low | Low | Low | Moderate |
| Guillamón-Escudero (2020) | High | Low | High | Low | Low | Low | Low | Low | Low | Low | Low |
| Wang (2018) | High | High | High | Low | Low | Low | Low | Low | Low | Low | Moderate |
| Nasimi (2019) | Low | Low | Low | Low | Low | Low | Low | Low | Low | Low | Low |
| Lee (2015) | High | High | High | Low | Low | Low | Low | Low | Low | Low | Moderate |
| Zengin (2021) | Low | Low | Low | Low | Low | Low | Low | Low | Low | Low | Low |
| Chang (2021) | High | High | High | Low | Low | Low | Low | Low | Low | Low | Moderate |
| Reijnierse (2015) | High | High | High | Low | Low | Low | Low | Low | Low | Low | Moderate |
| Tramontano (2016) | High | High | High | Low | Low | Low | Low | Low | Low | Low | Moderate |
| McIntosh (2013) | High | High | High | Low | Low | Low | Low | Low | Low | Low | Moderate |

Low = low risk of bias High = high risk of bias Moderate = moderate risk of bias

Criteria

External validity:

Q1. Was the study’s target population a close representation of the national population in relation to relevant variables?

Q2. Was the sampling frame a true or close representation of the target population?

Q3. Was some form of random selection used to select the sample, OR was a census undertaken?

Q4. Was the likelihood of nonresponse bias minimal?

Internal validity:

Q5. Were data collected directly from the subjects (as opposed to a proxy)?

Q6. Was an acceptable case definition used in the study?

Q7. Was the study instrument that measured the parameter of interest shown to have validity and reliability?

Q8. Was the same mode of data collection used for all subjects?

Q9. Was the length of the shortest prevalence period for the parameter of interest appropriate?

Q10. Were the numerator(s) and denominator(s) for the parameter of interest appropriate?

Q11. Summary item on the overall risk of study bias.

**Table S5** The details of diagnostic criteria and cut-off points of each study in general populations

| Low muscle mass | Diagnosis criteria | References |
| --- | --- | --- |
| BIA | 1. AWGS 2014^4^ or AWGS 2019^5^: SMI<7.0 kg/m^2^ for men and SMI<5.7 kg/m^2^ for women | Nasimi (2019), Yamada (2019), Chang (2017) |
|  | 1. EWGSOP 2010^6^ Rolland et al. (2009)^7^: SMI<6.75 kg/m^2^ for men and SMI<5.07 kg/m^2^ for women | Yamada (2013) |
|  | 1. IWGS 2011^8^: SMI<7.23 kg/m^2^ for men and SMI<5.67 kg/m^2^ for women | Tramontano (2016) |
|  | 1. EWGSOP 2010^6^ Schutz et al. (2002)^9^: FFMI< 18.9 kg/m^2^ for men, <15.4 kg/m^2^ for women | McIntosh (2013) |
| DXA | 1. AWGS 2014^4^ or AWGS 2019^5^: SMI < 7.0 kg/m^2^ for men and SMI < 5.4 m/s for women | Lee (2015), Wang (2018), Pal (2020) |
|  | 1. EWGSOP2 2018^10^: SMI < 7.0 kg/m^2^ for men and SMI < 5.5 m/s for women | Zengin (2021), Pal (2020) |
|  | 1. EWGSOP 2010^6^: SMI < 7.23 kg/m^2^ for men and SMI < 5.67 m/s for women | Reijnierse (2015) |
|  | 1. EWGSOP 2010^6^ Baumgartner et al. (1998)^11^: SMI < 7.26 kg/m^2^ for men and SMI < 5.45 m/s for women | Reijnierse (2015) |
|  | 1. EWGSOP 2010^6^ Delmonico et al. (2007)^12^: SMI < 7.25 kg/m^2^ for men and SMI < 5.67 m/s for women | Reijnierse (2015) |
| Equation | 1. Kyle et al. (2003)^13^. ASMM = -4.211 + (0.267*height2 / resistance) + (0.095*weight) + (1.909*sex (men = 1, women = 0)) + (-0.012*age) + (0.058*reactance) | Guillamón-Escudero (2020) |
| Low grip strength |  |  |
| HGS | 1. EWGSOP2 2018^10^: < 27 kg for men and 16 kg for women | Guillamón-Escudero (2020),  Zengin (2021), Pal (2020) |
|  | 1. AWGS 2014^4^: <26 kg for men and <18 kg for women | Lee (2015), Nasimi (2019), Wang (2018), Yamada (2019), Chang (2017) |
|  | 1. EWGSOP 2010^6^: < 30 kg for men and 20 kg for women | Yamada (2013), McIntosh (2013), Reijnierse (2015) |
|  | 1. AWGS 2019^5^ : <28 kg for men and <18 kg for women | Pal (2020) |
|  | 1. Lauretani et al. (2003**)**^14^ <30.3 kg for men and <19.3 kg for women | Reijnierse (2015) |
| Low physical performance |  |  |
| 4m GS | 1. EWGSOP2 2018^10^: GS < 0.8 m/s | Guillamón-Escudero (2020) |
|  | 1. AWGS 2014^4^: GS < 0.8 m/s | Lee (2015), Nasimi (2019) |
|  | 1. IWGS 2011^8^: GS < 1.0 m/s | Tramontano (2016) |
| 5m GS | 1. EWGSOP 2010^6^ Kressig et al. (2006)^15^ or AWGS 2014^4^: GS < 0.8 m/s | Yamada (2019),  McIntosh (2013) |
| 10 m GS | 1. EWGSOP 2010^6^: GS < 0.8 m/s | Yamada (2013) |
| 6 min-WT | 1. AWGS 2014^4^: GS < 0.8 m/s | Wang (2018) |

Abbreviations: ASMM: appendicular skeletal muscle mass; AWGS, Asian Working Group for Sarcopenia; BIA, bioelectrical impedance analysis; DXA, dual-energy X-ray absorptiometry; EWGSOP, European Working Group on Sarcopenia in Older People; EWGSOP2: European Working Group on Sarcopenia in Older People 2; FFMI, fat-free mass index; HGS, hand grip strength; IWGS, International Working Group on Sarcopenia; GS, gait speed; SMI, skeletal muscle mass index; 6 min-WT, 6 min-walk test.

**Figure S1** The Egger’s funnel plot of total CVDs in the meta-analysis using Egger’s test


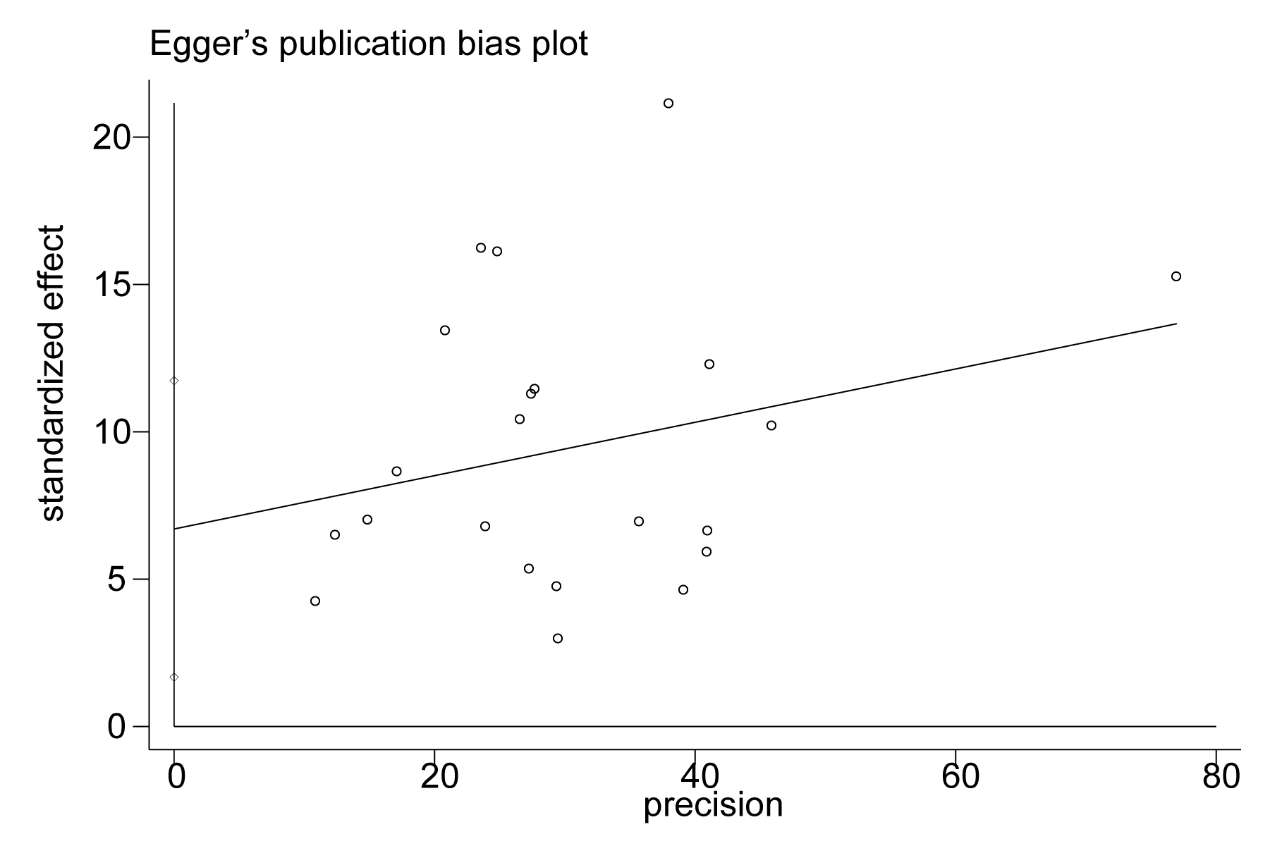


**Figure S2** The Egger’s funnel plot of total CVDs by the fill-and-trim method

**
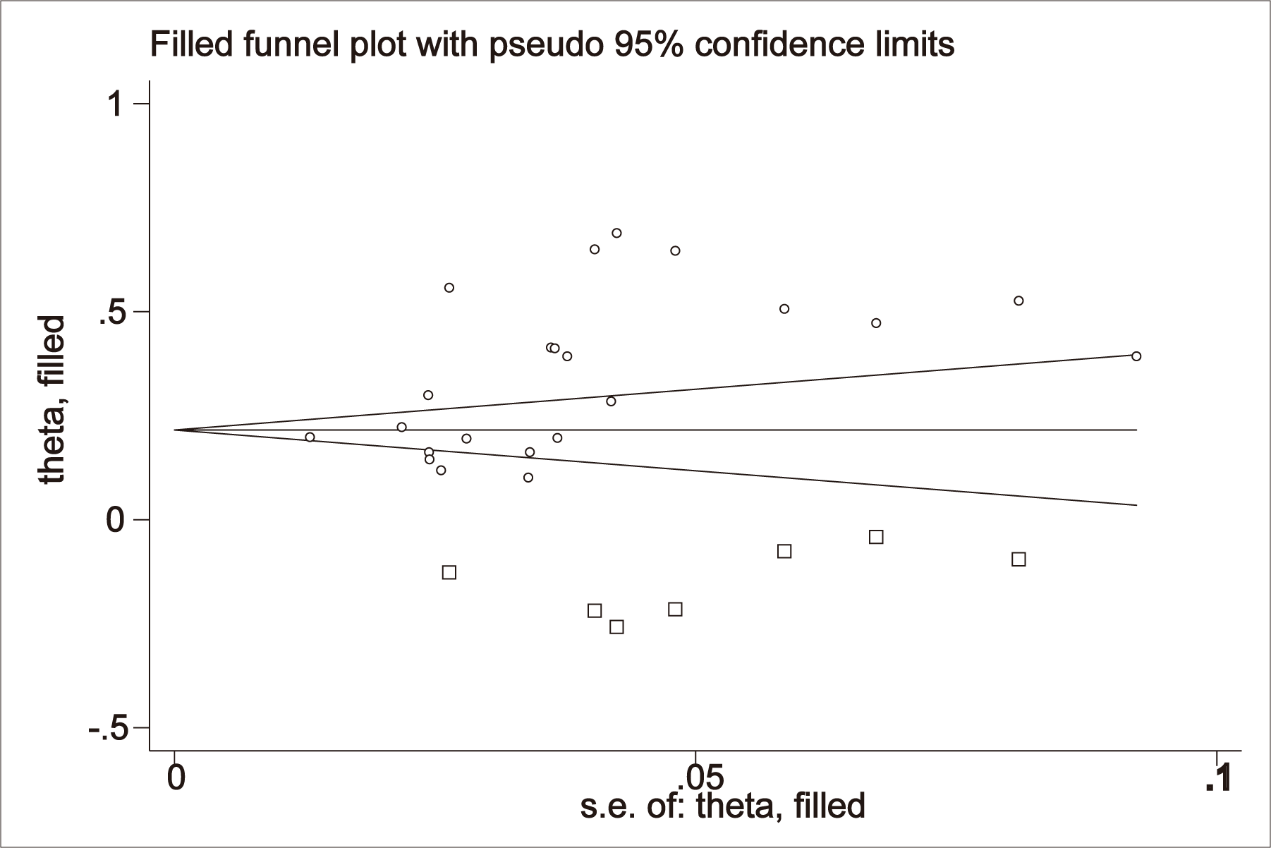
**

**Figure S3** The Egger’s funnel plot of ADHF, CAD and CHD studies in the meta-analysis using Egger’s test


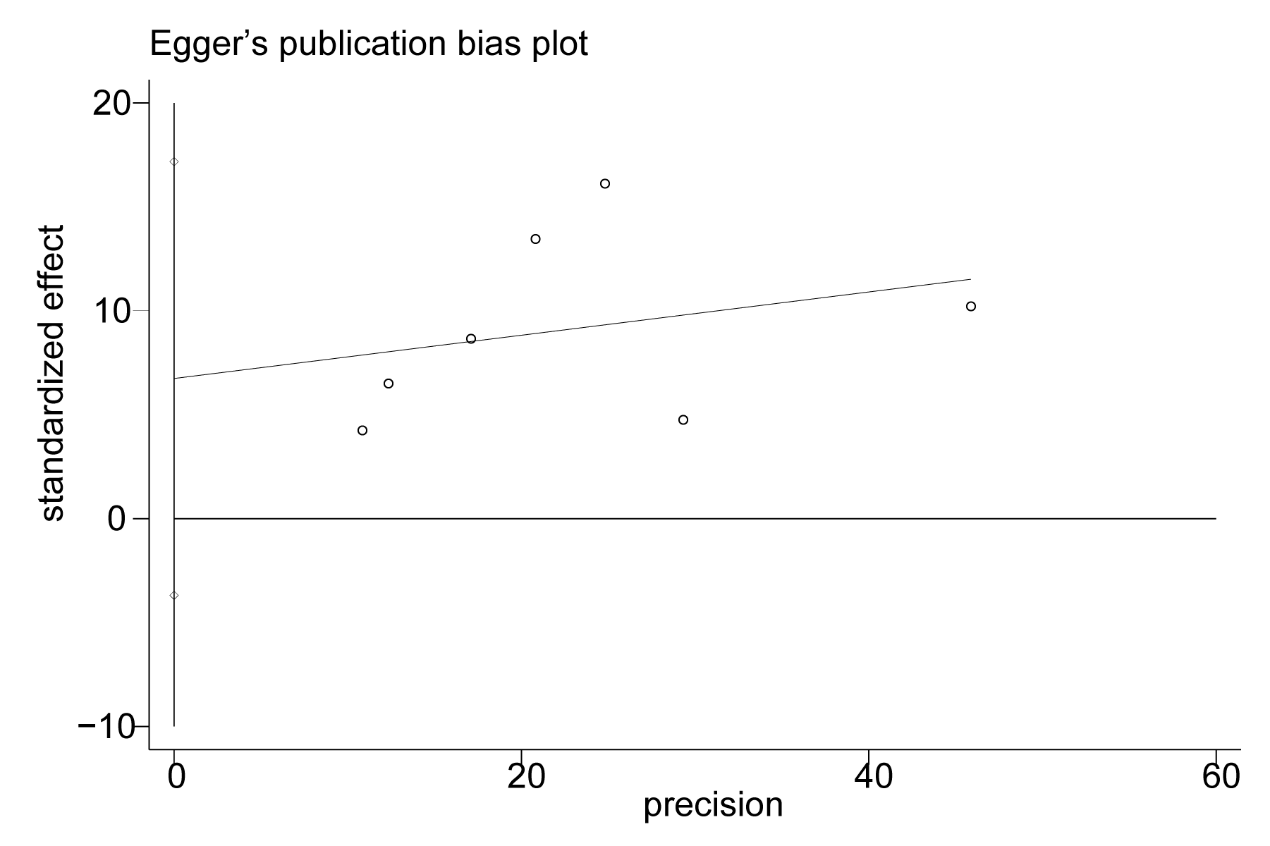


**Figure S4** The Egger’s funnel plot of CHF, CA and unclassed CVDs studies in the meta-analysis using Egger’s test

**
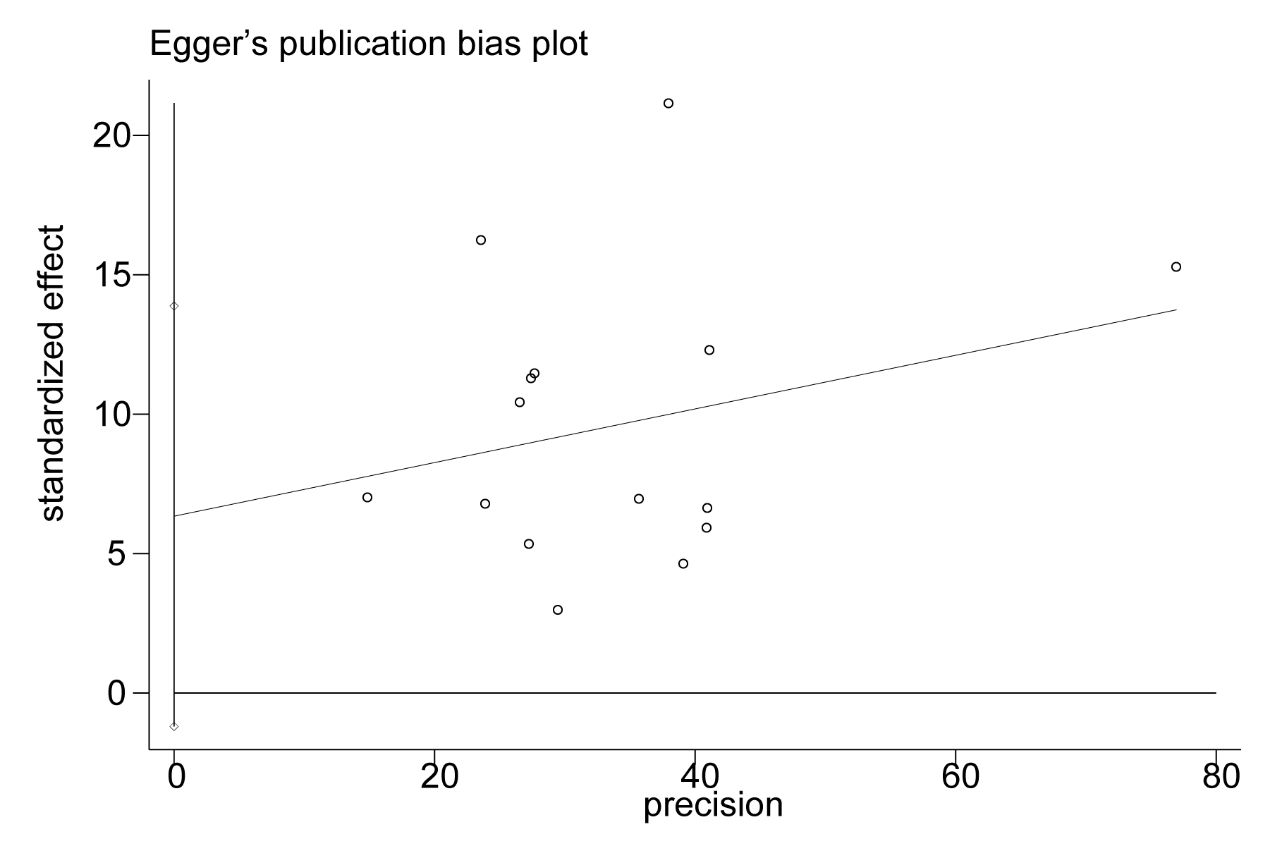
**

**Figure S5** Sensitivity analysis for the effect of individual studies (given named study in the Y axis is omitted) on the pooled prevalence of sarcopenia in CVDs. CI, confidence interval


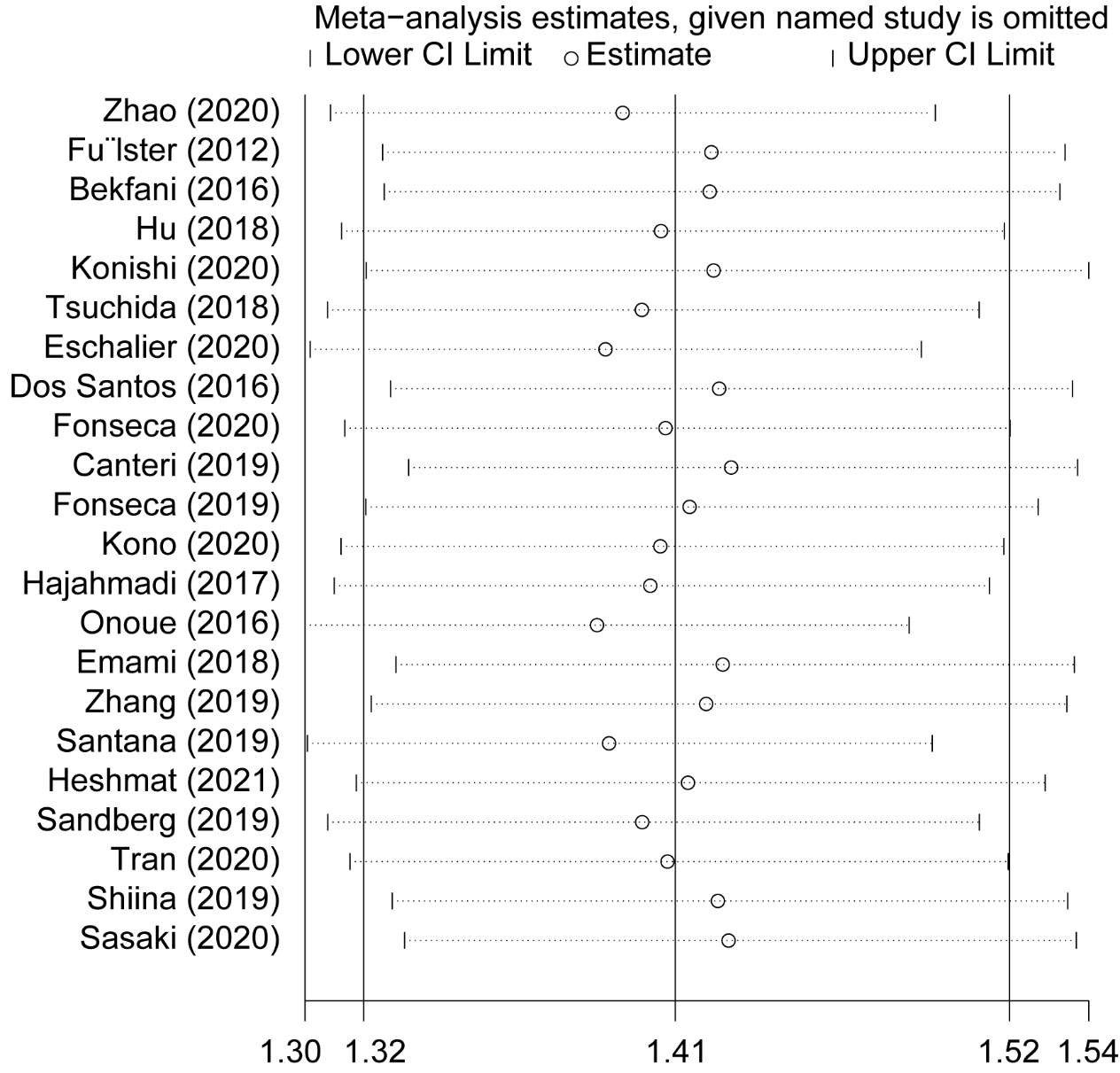


**Figure S6** The Egger’s funnel plot of general population in the meta-analysis using Egger’s test


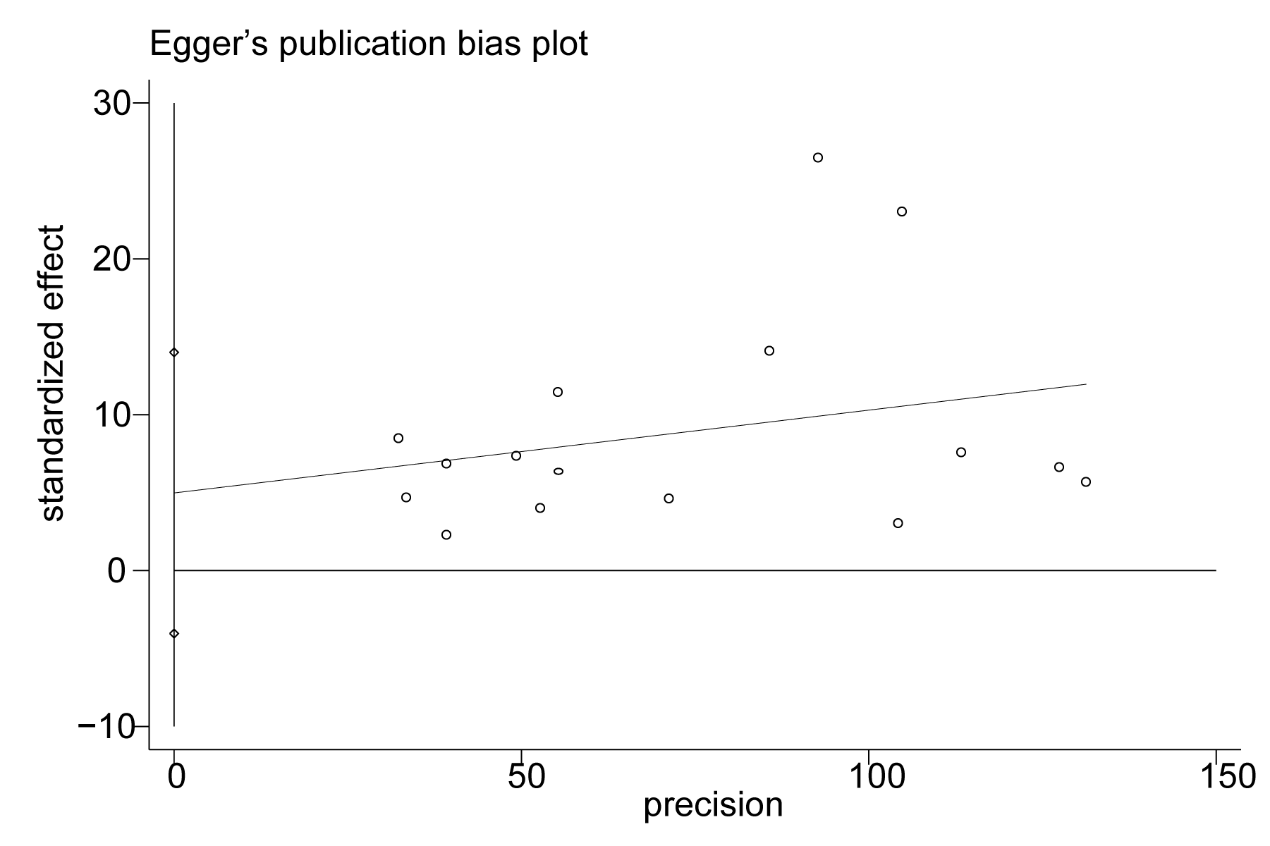


**Figure S7** Sensitivity analysis for the effect of individual studies (given named study in the Y axis is omitted) on the pooled prevalence of sarcopenia in general populations. CI, confidence interval


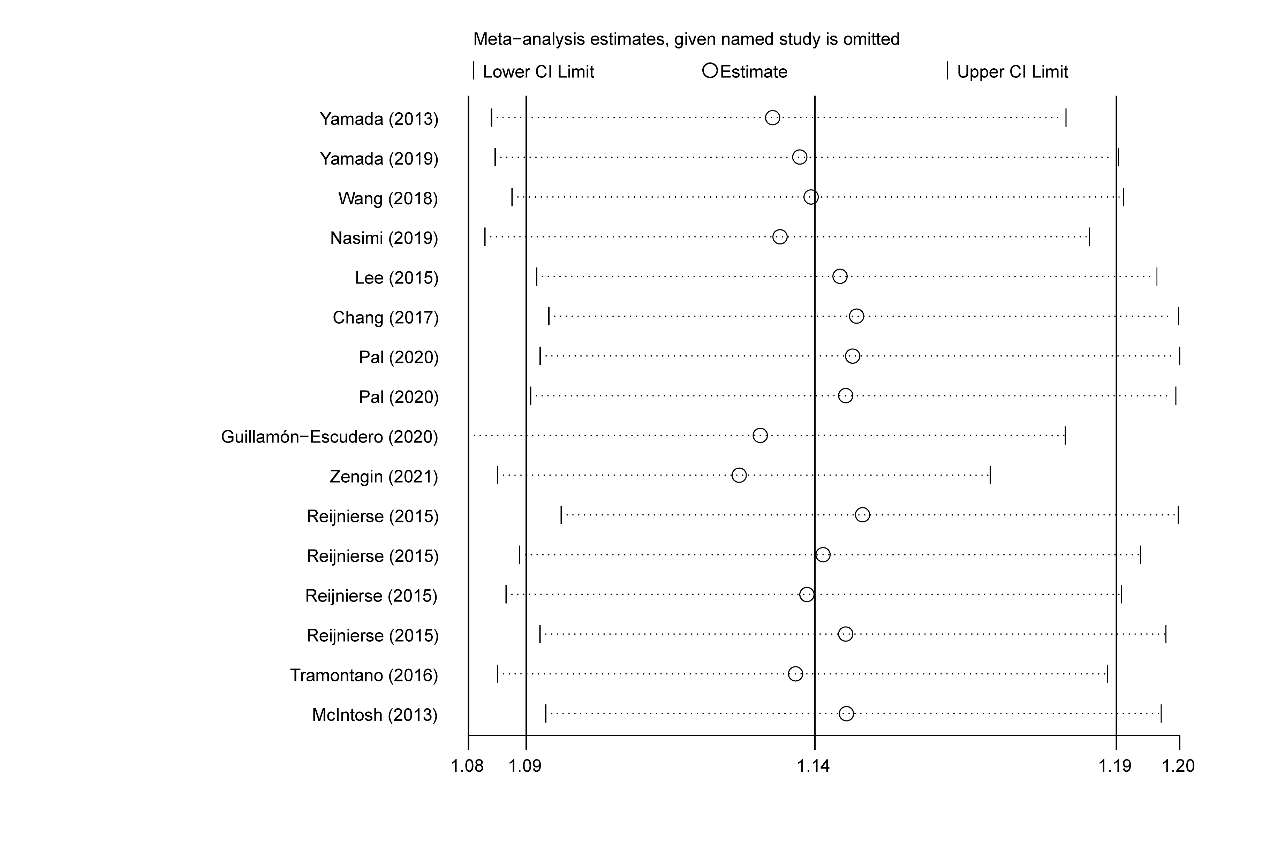


**References**

1. Pal R, Aggarwal A, Singh T, Sharma S, Khandelwal N, Garg A, et al. Diagnostic cut-offs, prevalence, and biochemical predictors of sarcopenia in healthy Indian adults: The Sarcopenia-Chandigarh Urban Bone Epidemiological Study (Sarco-CUBES). *Eur Geriatr Med*, 2020; **11**: 725-736.

2. Guillamon-Escudero C, Diago-Galmes A, Tenias-Burillo JM, Soriano JM, Fernandez-Garrido JJ. Prevalence of Sarcopenia in Community-Dwelling Older Adults in Valencia, Spain. *Int J Environ Res Public Health*, 2020; **17**.

3. Zengin A, Kulkarni B, Khadilkar AV, Kajale N, Ekbote V, Tandon N, et al. Prevalence of Sarcopenia and Relationships Between Muscle and Bone in Indian Men and Women. *Calcif Tissue Int*, 2021; **109**: 423-433.

4. Chen LK, Liu LK, Woo J, Assantachai P, Auyeung TW, Bahyah KS, et al. Sarcopenia in Asia: consensus report of the Asian Working Group for Sarcopenia. *J Am Med Dir Assoc*, 2014; **15**: 95-101.

5. Chen LK, Woo J, Assantachai P, Auyeung TW, Chou MY, Iijima K, et al. Asian Working Group for Sarcopenia: 2019 Consensus Update on Sarcopenia Diagnosis and Treatment. *J Am Med Dir Assoc*, 2020; **21**: 300-307 e2.

6. Cruz-Jentoft AJ, Baeyens JP, Bauer JM, Boirie Y, Cederholm T, Landi F, et al. Sarcopenia: European consensus on definition and diagnosis: Report of the European Working Group on Sarcopenia in Older People. *Age Ageing*, 2010; **39**: 412-23.

7. Rolland Y, Lauwers-Cances V, Cristini C, Abellan van Kan G, Janssen I, Morley JE, et al. Difficulties with physical function associated with obesity, sarcopenia, and sarcopenic-obesity in community-dwelling elderly women: the EPIDOS (EPIDemiologie de l'OSteoporose) Study. *Am J Clin Nutr*, 2009; **89**: 1895-900.

8. Fielding RA, Vellas B, Evans WJ, Bhasin S, Morley JE, Newman AB, et al. Sarcopenia: an undiagnosed condition in older adults. Current consensus definition: prevalence, etiology, and consequences. International working group on sarcopenia. *J Am Med Dir Assoc*, 2011; **12**: 249-56.

9. Schutz Y, Kyle UUG, Pichard C. Fat-free mass index and fat mass index percentiles in Caucasians aged 18-98 y. *Int J Obes Relat Metab Disord*, 2002; **26**: 953-960.

10. Cruz-Jentoft AJ, Bahat G, Bauer J, Boirie Y, Bruyere O, Cederholm T, et al. Sarcopenia: revised European consensus on definition and diagnosis. *Age Ageing*, 2019; **48**: 16-31.

11. Baumgartner RN, Koehler KM, Gallagher D, Romero L, Heymstleld SB, Ross RR, et al. Epidemiology of Sarcopenia among the Elderly in New Mexico. *Am J Epidemiol*, 1998; **147**: 755-63.

12. Delmonico MJ, Harris TB, Lee JS, Visser M, Nevitt M, Kritchevsky SB, et al. Alternative definitions of sarcopenia, lower extremity performance, and functional impairment with aging in older men and women. *J Am Geriatr Soc*, 2007; **55**: 769-74.

13. Kyle UG, Genton L, Hans D, Pichard C. Validation of a bioelectrical impedance analysis equation to predict appendicular skeletal muscle mass (ASMM). *Clin Nutr*, 2003; **22**: 537-43.

14. Lauretani F, Russo CR, Bandinelli S, Bartali B, Cavazzini C, Di Iorio A, et al. Age-associated changes in skeletal muscles and their effect on mobility: an operational diagnosis of sarcopenia. *J Appl Physiol (1985)*, 2003; **95**: 1851-60.

15. Kressig RW, Beauchet O, Group EGN. Guidelines for clinical applications of spatio-temporal gait analysis in older adults. *Aging Clin Exp Res*, 2006; **18**: 174-176.
